# Neuraminidase Activity Modulates Cellular Coinfection during Influenza A Virus Multicycle Growth

**DOI:** 10.1128/mbio.03591-22

**Published:** 2023-04-20

**Authors:** Zijian Guo, Yuanyuan He, Jian Xu, Ananya N. Benegal, Steven L. Brody, Michael D. Vahey

**Affiliations:** a Department of Biomedical Engineering, Washington University in St. Louis, St. Louis, Missouri, USA; b Center for Biomolecular Condensates, Washington University in St. Louis, St. Louis, Missouri, USA; c Division of Pulmonary and Critical Care Medicine, Department of Medicine, Washington University in St. Louis School of Medicine, St. Louis, Missouri, USA; Boston University School of Medicine

**Keywords:** influenza, neuraminidase, cellular coinfection, fluorescence microscopy

## Abstract

Infection of individual cells by multiple virions plays critical roles in the replication and spread of many viruses, but mechanisms that control cellular coinfection during multicycle viral growth remain unclear. Here, we investigate virus-intrinsic factors that control cellular coinfection by influenza A virus (IAV). Using quantitative fluorescence to track the spread of virions from single infected cells, we identify the IAV surface protein neuraminidase (NA) as a key determinant of cellular coinfection. We map this effect to NA’s ability to deplete viral receptors from both infected and neighboring uninfected cells. In cases in which viral infectious potential is low, genetic or pharmacological inhibition of NA increases the local spread of infection by increasing the viral load received by neighboring cells. These results identify virus-intrinsic factors that contribute to cellular multiplicity of infection and suggest that optimal levels of NA activity depend on the infectious potential of the virus in question.

## INTRODUCTION

Single cells infected by influenza A virus (IAV) can give rise to hundreds to thousands of new virions within a single replication cycle (1–3). These virions spread nonuniformly, producing wide variations in viral load that are concentrated around the initial site of infection. This phenomenon is widely recognized *in vitro*, providing the foundation for plaque assays for detecting and quantifying influenza and many other viruses. More recently, the development of reporter viruses (4–6) and genetic barcoding strategies (7, 8) have revealed that viral spread *in vivo* exhibits similar features: for example, showing sensitivity to anatomical compartmentalization (9, 10).

An important consequence of nonuniform viral spread is that it produces wide variation in the cellular multiplicity of infection (MOI). This has implications that are particularly important in the biology of IAV. The IAV genome is comprised of eight distinct RNA segments, and although most virions fail to deliver all eight segments individually (11, 12), complementation through coinfection can sustain productive infection (3). Cellular coinfection also enables reassortment when it occurs between distinct viral lineages (13); it enhances the replication of viruses that are poorly adapted to their hosts (14); and it can modulate cellular immune responses (15, 16). These wide-ranging contributions to influenza replication and spread make understanding the causes and consequences of IAV coinfection a high priority. However, the extent to which the frequency or degree of coinfection depends on specific properties of the virus or the target cell remains unclear.

Multiple factors may contribute to the spatial structure of viral spread and the degree of coinfection that occurs during multicycle growth. These include the number of virions released from infected cells (i.e., the burst size); the physical association between virions as they transit the extracellular environment; and the typical distance traveled by virions before they attach to and enter a naive cell (i.e., the degree of dispersal). Viral burst size can influence the frequency of coinfection by increasing the number of viral particles in the extracellular environment. Alternatively, viral aggregation (17), bacterial hitchhiking (18, 19), and clustered packaging into extracellular vesicles (20) have each been shown to enhance coinfection through the physical association of multiple infectious units. Finally, virion dispersal could influence the degree of coinfection by determining whether progeny virions remain concentrated at the site of infection or whether they spread out over a large range. While each of these considerations could influence the spread of IAV, specific links between IAV genotype and coinfection through these different routes have not been identified.

In the case of viral dispersal, the IAV surface proteins hemagglutinin (HA) and neuraminidase (NA) are likely to contribute (21–23). HA and NA are expressed on the surface of infected cells and packaged into virions, where they exhibit competing biochemical activities. NA cleaves the viral receptor sialic acid, allowing virus particles to spread throughout the host (24, 25). Released virions can initiate a new round of cellular infection through HA-mediated attachment to sialic acid on the surface of a naive cell (26). While both HA and NA are essential for virus replication and transmission, evolutionary data and laboratory experiments demonstrate that consequential mutations to one protein can be tolerated through compensatory mutations to the other (27–31). Biochemical data comparing HA avidity and NA catalytic activity for strains circulating in humans further supports the idea that efficient replication and transmission within a particular host depends on the balance between the two proteins’ competing activities (32, 33). However, the precise consequences of imbalanced HA and NA on viral spread—and whether other factors contribute to how well imbalance is tolerated—is not well understood. Further complicating this picture, some NAs can bind to sialic acid (34), and NAs cleave sialic acid both in *cis* (i.e., on the membrane of the infected cell) (35) and in *trans* (as in a standard enzyme-linked lectin assay [36]), raising the question of how these activities collectively contribute to virus release and attachment.

To begin addressing these questions, we investigated mechanistic links between the degree of coinfection that occurs during multicycle IAV growth, and the *in situ* activity of HA and NA on cells and viruses. Specifically, we investigated the localized spread and the degree of coinfection that occurs among virions originating from single sites of infection using high-resolution microscopy. We identify strain-specific differences in the degree of localized coinfection that different viruses support during multicycle growth in cell culture. We find that these differences are genetically linked to the NA segment, with the number of virions shed to neighboring cells varying inversely with NA enzymatic activity. We show that attenuation of NA activity using either genetic mutation or chemical inhibition can increase the spread of infection in viruses with high dependence on coinfection. Combined with stochastic modeling of virus spread, these results demonstrate the importance of viral adhesion and release in establishing the spatial structure of viral spread and suggest that the optimal balance between HA and NA activities for a given strain will depend on its infectious potential.

## RESULTS

### An imaging-based approach to tracking virion spread.

**FIG 1:**
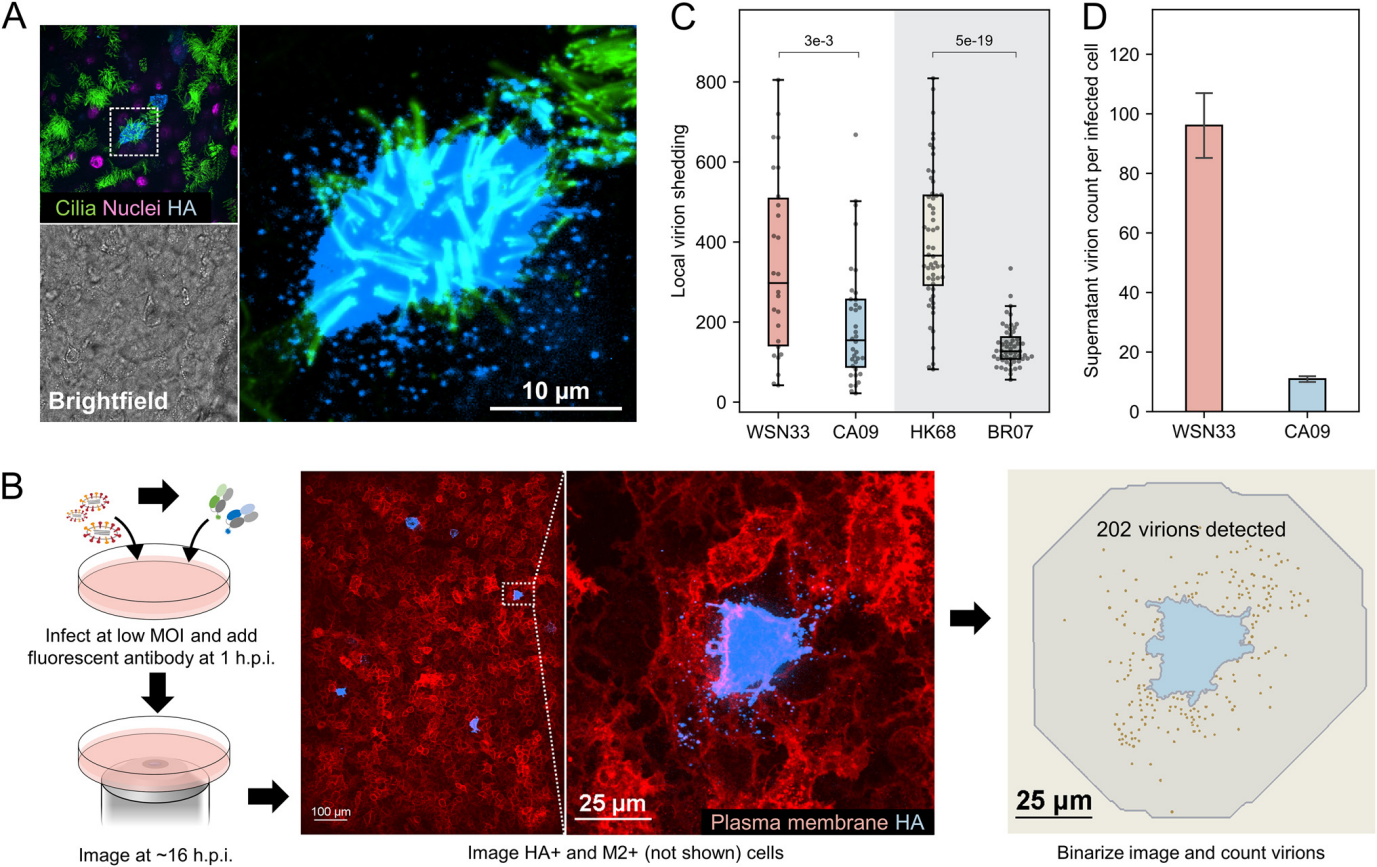
Virions produced by infected cells are preferentially shed to the nearest neighbors. (A) Localized virion spread in differentiated human tracheal epithelial cells (HTECs) cultured at the air-liquid interface following infection through the apical compartment. Contrast is exaggerated in the magnified image to show released virions. (B) Schematic and representative images illustrating the approach for measuring local virion shedding. Contrast in the hemagglutinin (HA) channel is exaggerated so that the shed virions are visible. (C) Quantification of local virion shedding for four influenza A virus (IAV) strains. Individual data points represent virions from single sites of infection. The data are combined from three biological replicates (at least nine sites of infection/replicate). The *P* values are determined by independent *t* tests. (D) Quantification of virions detected in the supernatant (average particle count per HA-positive cell) for WSN33 and CA09. The data are combined from three biological replicates (i.e., separate cell cultures infected and quantified in parallel). The error bars represent standard deviation. The *P* values are determined by independent *t* tests. MOI, multiplicity of infection.

Influenza A virus infection exhibits focal spread *in vivo*, in differentiated models of human airways (37), and in conventional cell culture. To determine how these observations translate to the spread of physical virus particles, we used fluorescence imaging to monitor the local spread of individual virions from infected cells. In primary human tracheal epithelial cells (HTECs) differentiated at the air-liquid interface, we observed large numbers of virions adjacent to infected cells at 16 h postinfection (h.p.i.), confirming that the local viral load can be high during secondary viral spread (Fig. 1A; Fig. S1).

10.1128/mbio.03591-22.1 FIG S1 Local virion spread in differentiated human tracheal cells. Images of differentiated human tracheal epithelial cells (HTECs) infected with different strains of influenza A virus (IAV) and imaged following fixation at 16 h.p.i. Hemagglutinin (HA) is labeled with CR9114 Fab; the cilia are visualized with an anti-acetyl tubulin antibody conjugated with Alexa Fluor 488. Contrast in the HA channel is exaggerated in the rightmost panels to make shed virions visible. Scale bars = 10 μm. Download FIG S1, PDF file, 3.8 MB. Copyright © 2023 Guo et al. 2023 Guo et al. https://creativecommons.org/licenses/by/4.0/ This content is distributed under the terms of the Creative Commons Attribution 4.0 International license.

These observations in differentiated HTECs, comprised of cells with motile cilia and others that secrete mucus, are qualitatively similar to the patterns of virion spread we observed in submerged cultures of A549 cells. We therefore proceeded to develop a fluorescence imaging-based approach to quantify the viral load per cell experienced within confluent A549 cell monolayers during secondary infection. This method allows us to determine the degree of localized coinfection caused by viruses derived from a common lineage, which therefore lack defined genetic differences. Following infection at low MOI, we incubate cells with fluorescently labeled Fab fragments that recognize conserved epitopes on HA away from the receptor-binding site. This allows us to image, segment, and count individual virions shed from sites of infection without disrupting the ability of these virions to bind to sialic acid (Sia) receptors. By blocking viral fusion with the endosome, Fabs derived from CR9114 (38) and FI6v3 (39) restrict replication to a single round. From these measurements, we define local virion shedding as the number of virions originating from isolated sites of infection (typically one single infected cell) that bind to and/or are internalized by neighboring uninfected cells (Fig. 1B). Volumetric imaging of infected A549 cells over time reveals that local virion shedding remains steady from 14 to 18 h postinfection (Fig. S2); we therefore selected the ~16-h time point for our data collection. Values for local virion shedding obtained in this way serve as a proxy for the degree of localized cellular coinfection that a particular viral strain supports during secondary infection.

10.1128/mbio.03591-22.2 FIG S2 Progression of virion shedding to neighboring cells over time. (A) Monitoring local virion shedding in A549 cells infected by WSN33. Confocal stacks of infected cells are taken starting from 12 h.p.i. Gray region marks the cell body, with detected virions highlighted in gold. The cells selected for analysis show expression of both HA and M2 on the cell surface. (B) Quantification of local virion shedding compiled from time series of seven cells infected by WSN33. The shaded region represents the 95% confidence interval. Download FIG S2, PDF file, 0.2 MB. Copyright © 2023 Guo et al. 2023 Guo et al. https://creativecommons.org/licenses/by/4.0/ This content is distributed under the terms of the Creative Commons Attribution 4.0 International license.

Using this approach, we first sought to measure the local spread of virions produced by different IAV strains and subtypes. We selected two H1N1 strains: A/WSN/1933 (WSN33) and A/California/04/2009 (CA09), and two H3N2 strains: A/Hong Kong/1/1968 (HK68) and A/Brisbane/10/2007 (BR07). These viruses represent a combination of lab-adapted and contemporary strains for the two IAV subtypes currently circulating in humans. We find that the number of virions shed to neighboring uninfected cells differs markedly both within a single strain and between strains (Fig. 1C), consistent with extreme heterogeneity in the outcome of IAV infection at low MOI (12), and suggesting that genetic differences between strains and subtypes contribute to localized virion spread.

In addition to the virus taken up by neighboring cells within the cell monolayer, infected cells are also expected to shed virus into the culture supernatant. This virus may become lost or neutralized, or it may travel long distances before entering a naive cell. To compare the local spread of virions to those released into the media, we counted the total number of virions released in the supernatant, normalized to the number of infected cells (Fig. S3). We find that during a single round of replication at an MOI of ~0.2, we detect an average of ~100 virions produced by each WSN33-infected cell and an average of ~10 virions from each CA09-infected cell (Fig. 1D). Consistent with our measurements of local virion shedding, these results demonstrate that these two H1N1 strains differ widely in the number of particles they produce per infected cell. However, for both strains, we detected more virus per cell adjacent to sites of infection than we detected in the media, supporting the conclusion that local cell-to-cell transmission is an important (but not the exclusive) pathway for the spread of infection.

10.1128/mbio.03591-22.3 FIG S3 Quantification of viral release into the culture supernatant. (A) Schematic showing the experimental procedure. The cells are infected (multiplicity of infection [MOI] = ~0.2) and labeled with fluorescent Fab starting at 1 h.p.i. The virus is collected at 16 h.p.i. and bound under centrifugation to the surface of a fresh A549 monolayer for quantification by confocal microscopy. (B) Quantification via Western blot of virus remaining in the supernatant following centrifugation onto A549 monolayers as in panel A. After spinning, 23% (WSN33) and 21% (CA09) of virions remain, suggesting that we are able to detect the majority of virions released into the supernatant. Download FIG S3, PDF file, 0.2 MB. Copyright © 2023 Guo et al. 2023 Guo et al. https://creativecommons.org/licenses/by/4.0/ This content is distributed under the terms of the Creative Commons Attribution 4.0 International license.

### Neuraminidase activity modulates local virion shedding.

**FIG 2:**
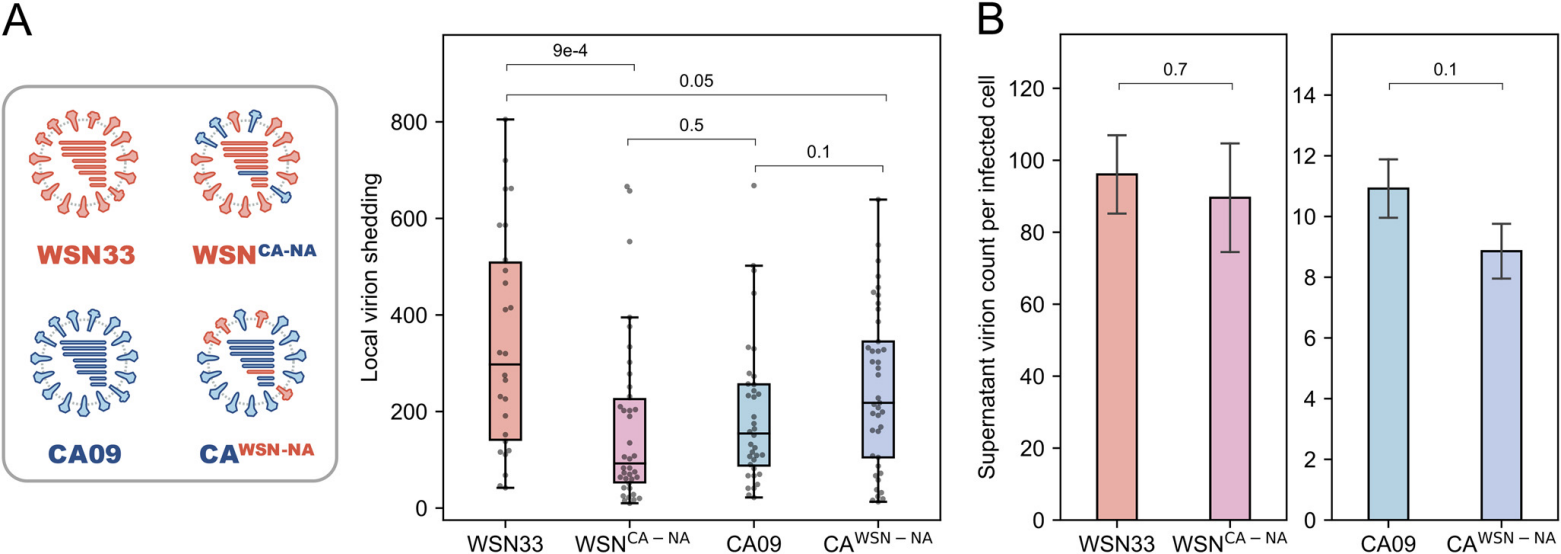
Neuraminidase activity affects local virion shedding without significantly changing shedding to the supernatant. (A) Schematic of neuraminidase (NA)-swap strains and their quantification of local virion shedding. The wild-type results presented previously are shown for comparison. The data are combined from three biological replicates (at least nine sites of infection/replicate). The *P* values are determined by independent *t* tests. (B) Comparison of virions detected in the supernatant (average per HA-positive cell) between wild-type and NA-swap strains. The data are combined from three biological replicates. Error bars represent standard deviation. The *P* values are determined by independent *t* tests.

To understand factors that could influence the local spread of virions, we hypothesized that the activity of neuraminidase (NA) may play an outsized role. NA depletes cell-surface sialic acids (35), reducing the chance of superinfection (40) and promoting viral release (25). To test whether NA contributes to differences in local virion shedding observed between strains, we created recombinant viruses in which the NA segment of CA09 and WSN33 were exchanged (Fig. 2A, left). Expression of WSN33 NA in a CA09 genetic background modestly increased local virion shedding (although not to a statistically significant extent; *P = *0.1), while expression of CA09 NA in a WSN33 background significantly decreased local virion spread (Fig. 2A, right). To determine whether exchanging NA segments between these two strains also changed the potential for long-distance virion spread, we quantified virion release into the culture supernatant per infected cell. The number of virions detected in the supernatant remained similar following the exchange of NA segments (Fig. 2B). Thus, CA09 NA expressed in a WSN33 background decreases the proportion of virions that spread adjacent to the initial site of infection, whereas WSN33 NA expressed in a CA09 background increases the proportion of virions that remain localized at the site of infection. These results establish local virion shedding as a viral phenotype that differs between strains and depends in part on the NA segment.

### The *in situ* activity of NA against cell-surface Sia differs between strains.

**FIG 3:**
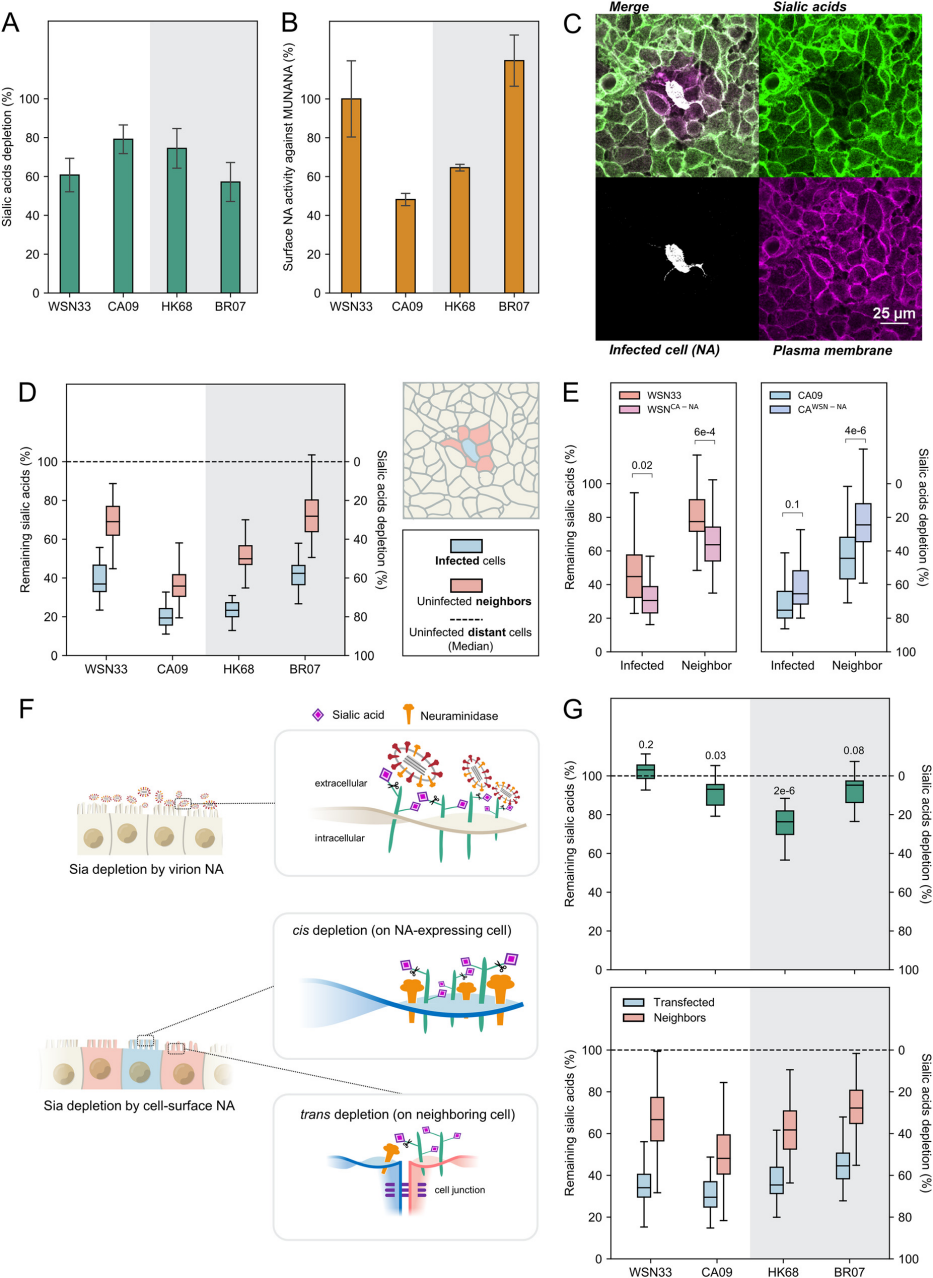
Cell-surface NA depletes sialic acid (Sia) in *cis* and in *trans*. (A) Cell-surface Sia depletion on cells infected by each viral strain at 16 h postinfection (h.p.i.). Quantifications are based on HA-positive cells. A value of 100% corresponds to complete depletion. (B) Surface NA activity against 2′-(4-methylumbelliferyl)-α-d-*N*-acetylneuraminic acid (MUNANA), normalized to data for WSN33. The experimental details are provided in the Materials and Methods section under “Quantification of NA activity with MUNANA.” (C) Image showing Sia, cell-surface NA, and plasma membrane (labeled with CellMask Orange) in the proximity of an isolated cell infected with HK68 at 16 h.p.i. (D) Quantification of remaining Sia on the surface of infected cells and uninfected neighbors at 16 h.p.i. The cells were selected based on NA expression by incubating with the anti-NA antibody 1G01 immediately before imaging. The data are combined from three biological replicates (at least eight sites of infection/replicate). (E) Quantification of remaining Sia on the surface of infected cells (determined by HA expression) and uninfected neighbors for viruses with exchanged NA segments at 16 h.p.i. The data are combined from three biological replicates (at least 14 sites of infection/replicate). The *P* values are determined by independent *t* tests. (F) Illustration of Sia depletion by virion-associated NA (top) and cell-associated NA (bottom). (G, top) Quantification of Sia depletion following incubation with virions. The results are normalized to Sia levels on untreated cell surfaces. The *P* values are determined from independent *t* tests relative to the untreated group. (Bottom) Quantification of Sia depletion by cell-surface NA (delivered through transfection, imaged at 48 h after transfection). The cells were selected based on NA expression. The data are combined from three biological replicates (at least 24 cells/replicate).

NA could contribute to local virion shedding in multiple ways: increasing or decreasing viral adhesion depending on its enzymatic activity or influencing virion assembly through unrelated mechanisms. To help distinguish between these possibilities, we first sought to compare the ability of different NAs to deplete sialic acid from the cell surface. We labeled sialic acid on A549 monolayers infected at low MOI (~0.003) using mild periodate oxidation followed by conjugation with an aldehyde-reactive fluorophore (41) (Fig. S4 and S5). Comparing Sia levels across cells infected with different strains provides a metric for the *in situ* activity of NA against its native substrates in cell culture. These measurements show that the selected strains deplete Sia to different extents (Fig. 3A) and that the efficiency with which NA removes Sia from the cell surface does not generally correlate with its activity against the small-molecule substrate 2′-(4-methylumbelliferyl)-α-d-*N*-acetylneuraminic acid (MUNANA) (Fig. 3B), consistent with prior work evaluating NA activity against larger, multiply sialylated substrates (42–44).

10.1128/mbio.03591-22.4 FIG S4 Quantifying depletion of cell-surface sialic acid (Sia) using hydrazide coupling. (A) Image showing regions of interest (ROIs) sampled from the surface of an infected cell (HA+), uninfected neighbors, and uninfected distant cells. (B) Enlarged ROIs in split view. The number on each split channel represents the mean intensity. The number on the Merge channel represents the Sia signal normalized by the plasma membrane (PM) signal, proportional to Sia per unit membrane area. The size of each ROI is 0.55 μm × 0.55 μm. Download FIG S4, PDF file, 0.2 MB. Copyright © 2023 Guo et al. 2023 Guo et al. https://creativecommons.org/licenses/by/4.0/ This content is distributed under the terms of the Creative Commons Attribution 4.0 International license.

10.1128/mbio.03591-22.5 FIG S5 Hydrazide conjugation sensitively and reproducibly reports Sia depletion by exogenous sialidase. Quantification of Sia levels on A549 cell monolayers treated with indicated concentrations of *Cp*NA for 30 min at 37°C using hydrazide coupling (left) and lectin labeling (S. nigra lectin [SNA]; right). The points represent mean values of three individual experiments (two for untreated group). The error bars show standard deviation. Download FIG S5, PDF file, 0.2 MB. Copyright © 2023 Guo et al. 2023 Guo et al. https://creativecommons.org/licenses/by/4.0/ This content is distributed under the terms of the Creative Commons Attribution 4.0 International license.

In addition to the depletion of Sia from the surface of infected cells (i.e., in *cis*), we also observe depletion from the surface of adjacent uninfected cells (i.e., in *trans*; Fig. 3C). *Trans* depletion of Sia follows similar trends across the strains tested as *cis* depletion (Fig. 3D) and also occurs in cells with greater apical-basal polarity (MDCK; Fig. S6). Incubation with 50 nM oseltamivir after transfection significantly reduced Sia depletion (Fig. S7), suggesting that this observation depends on NA activity. Viral strains with swapped NAs (WSN^CA-NA^ and CA^WSN-NA^) showed similar *cis* and *trans* Sia depletion as the strains from which their NAs were derived (Fig. 3E). We reasoned that Sia depletion in *trans* could be driven by NA on the surface of cells, released viruses, or both. To evaluate the contributions of these two sources of NA activity, we compared Sia depletion driven by virus-associated NA with that of NA expressed on the surface of cells through transfection in the absence of other viral proteins (“cell-associated” NA) (Fig. 3F). Virus-associated NA (added such that at an average of ~10 virions attach to each cell, as confirmed by confocal microscopy after incubation at 37°C for 1 h) shows a relatively modest effect on Sia depletion at this viral density, reaching a maximum of ~25% for HK68 (Fig. 3G, top), whereas cell-associated NA achieves similar *cis* and *trans* depletion of cell surface Sia as that observed in virus-infected cells (Fig. 3G, bottom). Although we cannot rule out the contributions from shed virions or other *trans*-acting mechanisms that could lead to a reduction in Sia on the surface of cells adjacent to sites of infection, these experiments demonstrate that both *cis* and *trans* Sia reduction are sensitive to NA inhibitors and that cell-surface NA in the absence of other viral factors is sufficient to recapitulate trends observed during viral infection.

10.1128/mbio.03591-22.6 FIG S6 *Cis* and *trans* depletion of Sia is observed following infection of multiple cell types. (A) Images showing HA, Sia, and plasma membrane in the proximity of cells infected with CA09 at a MOI of 0.003. (B) Quantification of remaining Sia on infected and neighboring cells for A549 and MDCK cell lines. The data are from at least 17 sites of infection/cell line. Download FIG S6, PDF file, 0.4 MB. Copyright © 2023 Guo et al. 2023 Guo et al. https://creativecommons.org/licenses/by/4.0/ This content is distributed under the terms of the Creative Commons Attribution 4.0 International license.

10.1128/mbio.03591-22.7 FIG S7 Quantification of Sia depletion under oseltamivir treatment. The cells were incubated for 48 h at 33°C after WSN33, CA09, HK68, and BR07 NA transfection. For oseltamivir groups, 50 nM oseltamivir was added at the time of transfection. All treatments were performed side by side for comparison and contain data from at least 10 cells. The *P* values were determined by independent *t* tests. Download FIG S7, PDF file, 0.1 MB. Copyright © 2023 Guo et al. 2023 Guo et al. https://creativecommons.org/licenses/by/4.0/ This content is distributed under the terms of the Creative Commons Attribution 4.0 International license.

### *In situ* NA activity shapes permissiveness to viral attachment in a strain-specific manner.

**FIG 4:**
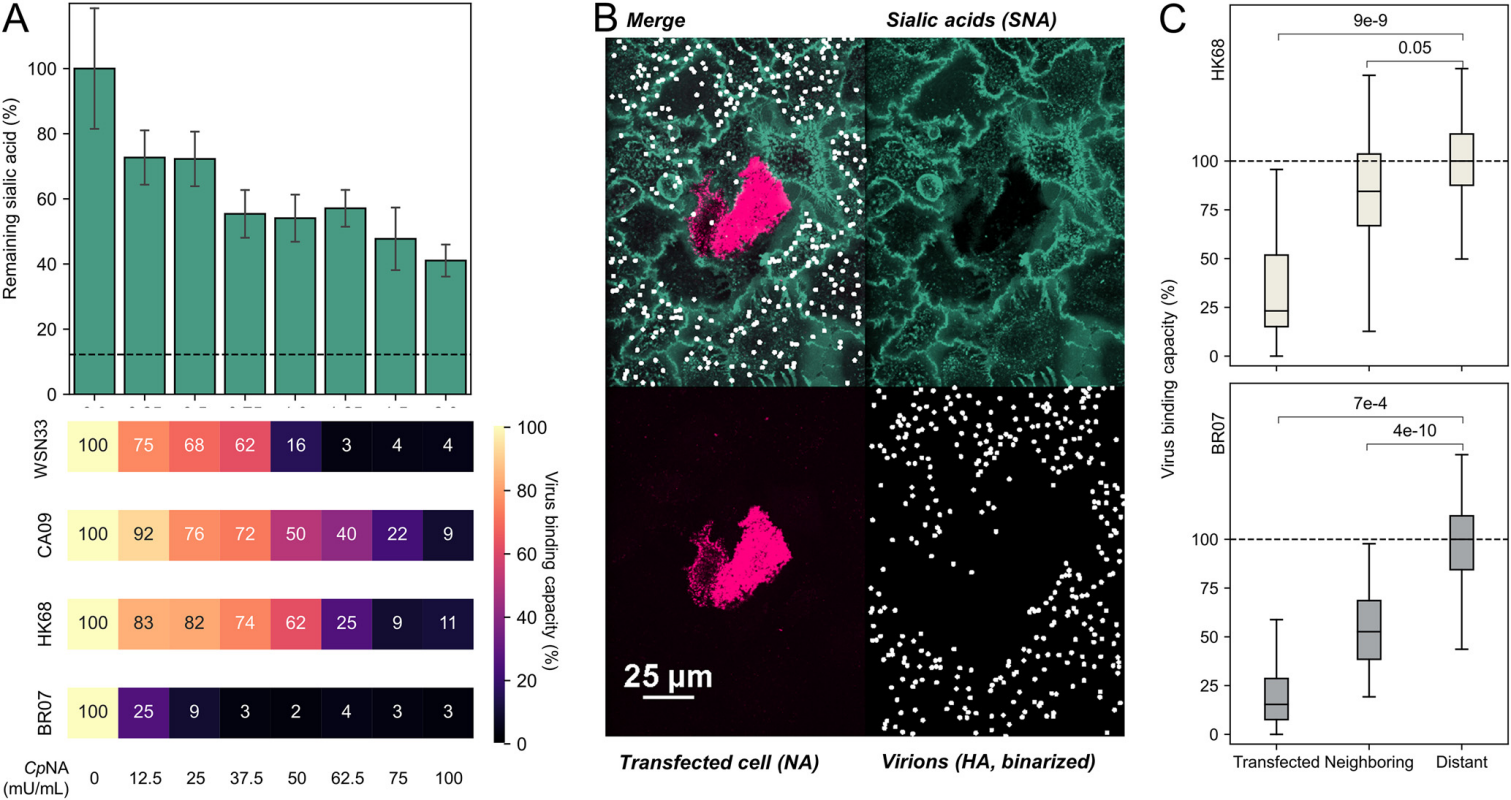
Sialic acid depletion reduces viral attachment in a strain-dependent manner. (A, top) Cell-surface Sia abundance following treatment with *Cp*NA. The concentrations are specified below. The dashed line indicates signal from CMP-sialic acid transporter SLC35A1 knockout cells treated with the highest *Cp*NA concentration, representing the background of Sia labeling reaction. (Bottom) Relative binding capacities for each viral strain following *Cp*NA treatment. The data are combined from three biological replicates. A replicate is defined by eight individual cell cultures treated by the indicated concentration of *Cp*NA. (B) Representative image showing virus attachment to cell monolayers with sparse expression of HK68 NA (virus strain BR07). (C) Quantification of virus binding capacity on HK68 NA-expressing (Transfected) cells, their adjacent cells (Neighboring), and other cells (Distant). The data for HK68 and BR07 virions, which show different binding avidity, are shown in the top and bottom panels. The data are from three biological replicates (at least 21 NA-expressing cells/replicate) and are normalized to the distant cell data. The *P* values are determined by independent *t* tests. SNA, S. nigra lectin.

To understand how Sia depletion affects the binding of virions, we compared virion attachment for the four strains from our initial test. The HAs of these viruses have different avidity for human receptors (33, 45), suggesting that they may differ in their sensitivity to Sia depletion. To determine how attachment of each viral strain to A549 monolayers changes following reduction of cell surface Sia, we first measured changes in virus binding following treatment with exogenous sialidase from Clostridium perfringens (*Cp*NA), an enzyme with broad specificity (46). The four strains respond differently to Sia depletion by *Cp*NA, with BR07 showing the greatest reduction even at modest levels of Sia depletion and HK68 and CA09 showing the most persistent binding as Sia levels are reduced (Fig. 4A). We observed similar trends using native viral NA; expression of HK68 NA in cells reduces attachment of both HK68 and BR07 virions to NA-expressing cells, as well as to neighboring cells in which Sia is depleted (Fig. 4B and C). Taken together, these results demonstrate that virus-associated HA influences the extent of virion attachment to cells, while cell-associated NA influences which cells are most permissive to attachment.

### Increased *in situ* NA activity reduces local cellular coinfection.

**FIG 5:**
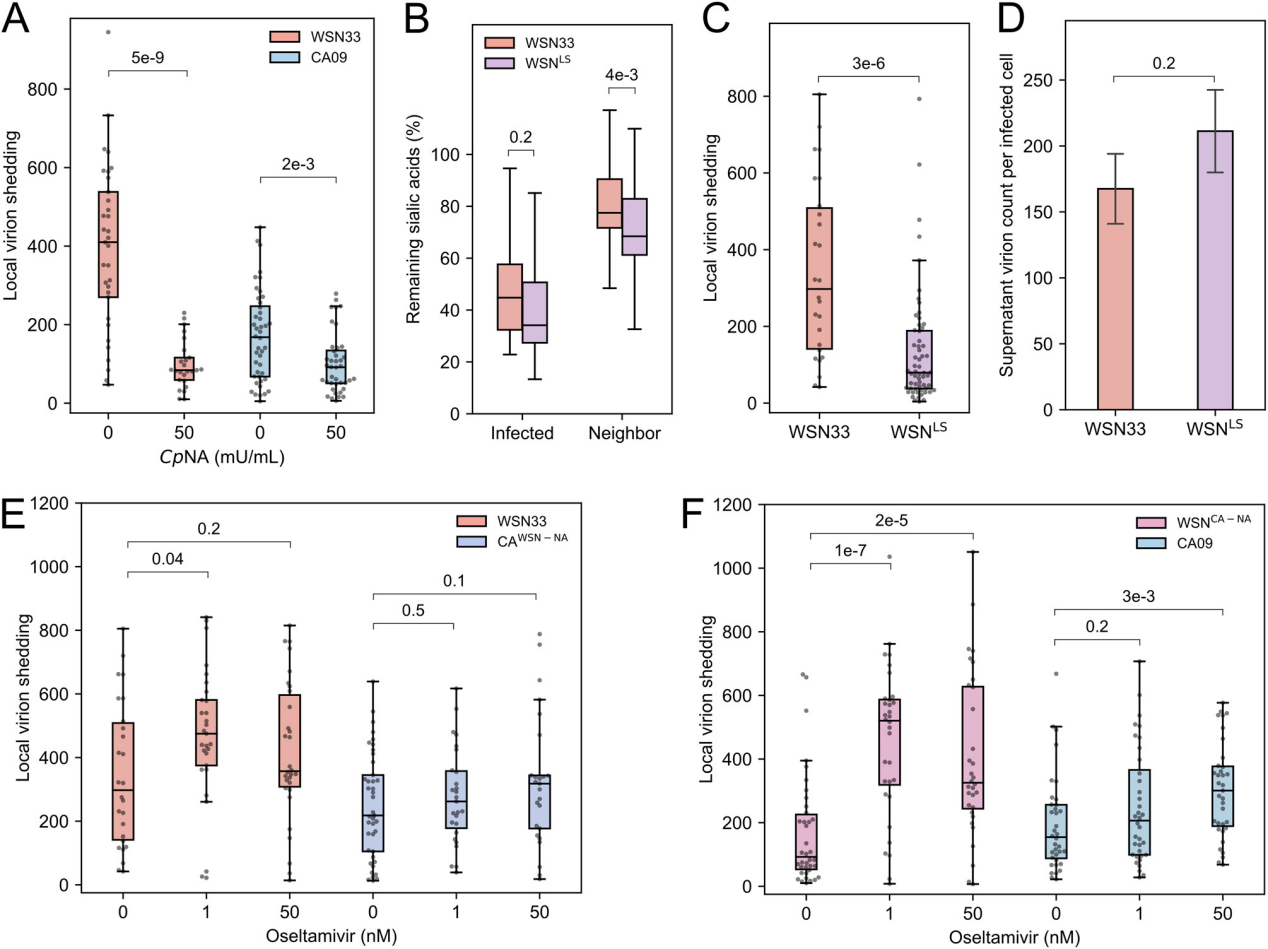
Decreasing NA activity increases local virion shedding. (A) Quantification of local virion shedding under continuous treatment with exogenous sialidase (*Cp*NA). The data are combined from three biological replicates (at least 10 sites of infection per replicate). The *P* values are determined from independent *t* tests. (B) Quantification of *cis* and *trans* cleavage of Sia by WSN33 and WSN^LS^. The data are combined from three biological replicates (at least five sites of infection/replicate). The *P* values are determined from independent *t* tests. (C) Quantification of local virion shedding for WSN33 and WSN^LS^. The data are combined from three biological replicates (at least 11 sites of infection/replicate). The *P* values are determined from independent *t* tests. (D) Comparison of virions detected in the supernatant (average per HA-positive cell) between WSN33 and WSN^LS^. The absolute value of WSN33 is not comparable to results shown previously as they were performed on different days. The data are combined from three biological replicates. The error bars represent standard deviation. The *P* values are determined by independent *t* tests. (E) Quantification of local virion shedding for viruses with WSN33 NA (in WSN33 or CA09 backgrounds) under oseltamivir treatment. The data are combined from three biological replicates (at least 10 sites of infection/replicate). The *P* values are determined from independent *t* tests. (F) Quantification of local virion shedding for viruses with CA09 NA (in WSN33 or CA09 backgrounds) under oseltamivir treatment. The data are combined from three biological replicates (at least 10 sites of infection/replicate). The *P* values are determined from independent *t* tests.

To determine whether direct cell-to-cell viral spread is sensitive to Sia depletion, we measured local virion shedding following treatment with exogenous sialidase (*Cp*NA). We observed an ~5-fold reduction in local virion shedding for WSN33-infected cells and an ~2-fold reduction for cells infected with CA09 (Fig. 5A), confirming that cell-to-cell viral spread is Sia-dependent. Based on these observations, we reasoned that high *in situ* NA activity (as exhibited by CA09 NA; Fig. 3A) could deplete Sia receptors from cells adjacent to the initial site of infection, reducing virus binding and uptake by these cells. This could potentially explain the differences in local virion shedding we observed when NA segments were swapped between WSN33 and CA09 (Fig. 2A).

We next perturbed the *in situ* NA activity of WSN33 by rescuing a virus (WSN^LS^) harboring a 16-residue insertion in the NA stalk that restores the length of contemporary N1 NAs and increases NA activity (47–49). This longer-stalk NA matches CA09 NA in overall size but preserves the catalytic domain of WSN33 NA. Consistent with prior comparisons of long- and short-stalked NAs (50–52), our *in situ* measurements show higher Sia depletion in *trans* by this strain (Fig. 5B). Viruses with long-stalk NA also show reduced local virion shedding relative to the parental strain (Fig. 5C), without showing reduction in virus released into the supernatant (Fig. 5D).

Although these results are consistent with a direct link between *in situ* NA activity and the extent of cellular coinfection that occurs during local viral spread, they do not rule out structural contributions from different NAs that may affect virus assembly. To specifically test how NA activity contributes to coinfection in the absence of genetic changes, we treated cells infected with viral strains harboring WSN33 NA and CA09 NA with various concentrations of the NA inhibitor oseltamivir (53). In contrast to strains harboring WSN33 NA, we found that oseltamivir treatment leads to a significant increase in local virion shedding in strains harboring CA09 NA (Fig. 5E and F). These differences in responsiveness may arise from the differential changes in Sia depletion we observe when these two strains are treated with oseltamivir (Fig. S7). Collectively, these results suggest that changes in NA activity—through either mutation or chemical inhibition—modulate the degree of local virion shedding that occurs during viral spread.

### Increased cellular MOI promotes the spread of CA09 infection.

**FIG 6:**
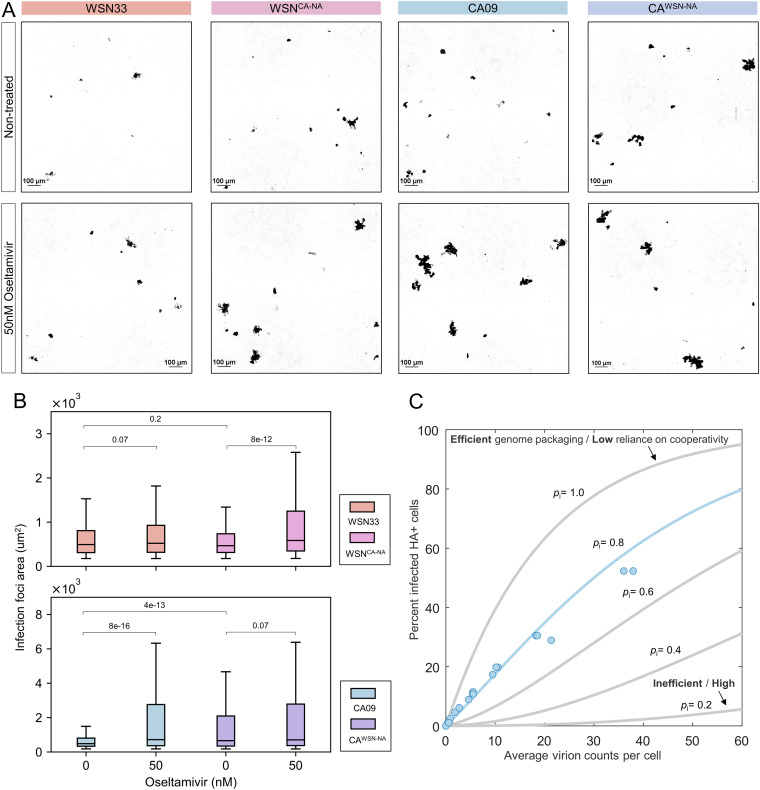
NA activity modulates the local spread of infection. (A) Representative images of infected clusters of A549 cells at 48 h.p.i. Infected cells (shown in black) are visualized by an M2-specific Fab. (B) Quantification of the size distribution of infection foci at 48 h.p.i. The data are combined from three biological replicates containing a total of 500 to 1,000 foci for each strain. The *P* values were determined by Kolmogorov-Smirnov tests. (C) Relating the average virion count per cell to the probability of infection. The experimental data for CA09 are shown as blue circles. Theoretical curves from an infection model (see Materials and Methods section) are shown in gray, with the best fit shown in blue. The probability of segment delivery (*p_i_*) varies as indicated, while the fraction of noninfectious virions (ϕ) remains constant (0.05). See also Fig. S8.

Localized viral spread and the high viral loads it supports may enhance infection by IAV, in which the ratio of total virus particles to fully infectious units is thought to range from ~10 to 100 (1, 54–56). These estimates generally do not account for the percentage of virions that attach to the cell surface and may overestimate the actual value. To determine how *in situ* NA activity contributes to the local spread of infection, we compared the size of infection foci for cells infected with WSN33 or CA09 during multicycle viral spread, as well as their counterparts with swapped NAs. As an additional perturbation of NA activity, we also tested the effects of treatment with oseltamivir. For viruses harboring CA09 NA, but not those with WSN33 NA, the size of infection foci increased significantly following NA inhibition (Fig. 6A and B). This follows the trends observed for these strains in local virion shedding, suggesting that increased particle counts in neighboring cells map to increased probability of localized secondary infection.

10.1128/mbio.03591-22.8 FIG S8 Modeling virus infection. (A) Illustration of virus populations represented by the model. Semi-infectious or fully infectious particles can have different probabilities of delivering each segment. The proportion of the virus population that contributes to infection is given by ϕ. (B) Predictions from the infection model for different proportions of noninfectious virions (1−ϕ) with *p_i_* = 0.8 (i.e., an infectious virus delivers any particular genome segment with an 80% probability). The results are shown for the percentage of HA+ cells. Fitting CA09 infectivity data suggest values of *p_i_* = 0.8 and ϕ = 0.05. (C) Predictions from the infection model for different segment delivery probabilities *p_i_*, where ϕ = 0.5. The S shapes of curves with lower *p_i_* values do not recapitulate data. Download FIG S8, PDF file, 0.3 MB. Copyright © 2023 Guo et al. 2023 Guo et al. https://creativecommons.org/licenses/by/4.0/ This content is distributed under the terms of the Creative Commons Attribution 4.0 International license.

Assuming that each infected cell is surrounded by 10 nearest neighbors, our data for CA09 imply that an increase from ~10 to ~20 virions/cell (obtained by dividing the data from Fig. 5F by the number of neighboring cells) significantly increases the probability of infection (Fig. 6A and B). To verify whether this is the case, we measured the infectious potential of CA09 by mapping the relationship between virion counts and infection in A549 cells. We observed that the proportion of infected cells increases linearly with the average numbers of virions/cell over a wide range (Fig. 6C). At an average of ~10 virions/cell, the proportion of infected cells remains modest, at around 20%. By fitting these data to an infection model in which all cells are equally susceptible (Fig. 6C; Fig. S8A; Materials and Methods), we find that its high linearity is consistent with a large proportion of noninfectious particles, combined with a small proportion of virions that are either semi- or fully infectious and that deliver individual genome segments with an efficiency of ~80% (Fig. S8B). In contrast, a large proportion of semi-infectious particles with less efficient genome delivery would exhibit higher cooperativity, producing an S-shaped curve in our model (Fig. S8C). These results confirm that infection of A549 cells by CA09 is inefficient at low particle counts but that the local spread of infection (as measured by the size of infection foci) can be increased by attenuating NA activity.

### The predicted optimal balance between IAV surface proteins depends on infectious potential.

**FIG 7:**
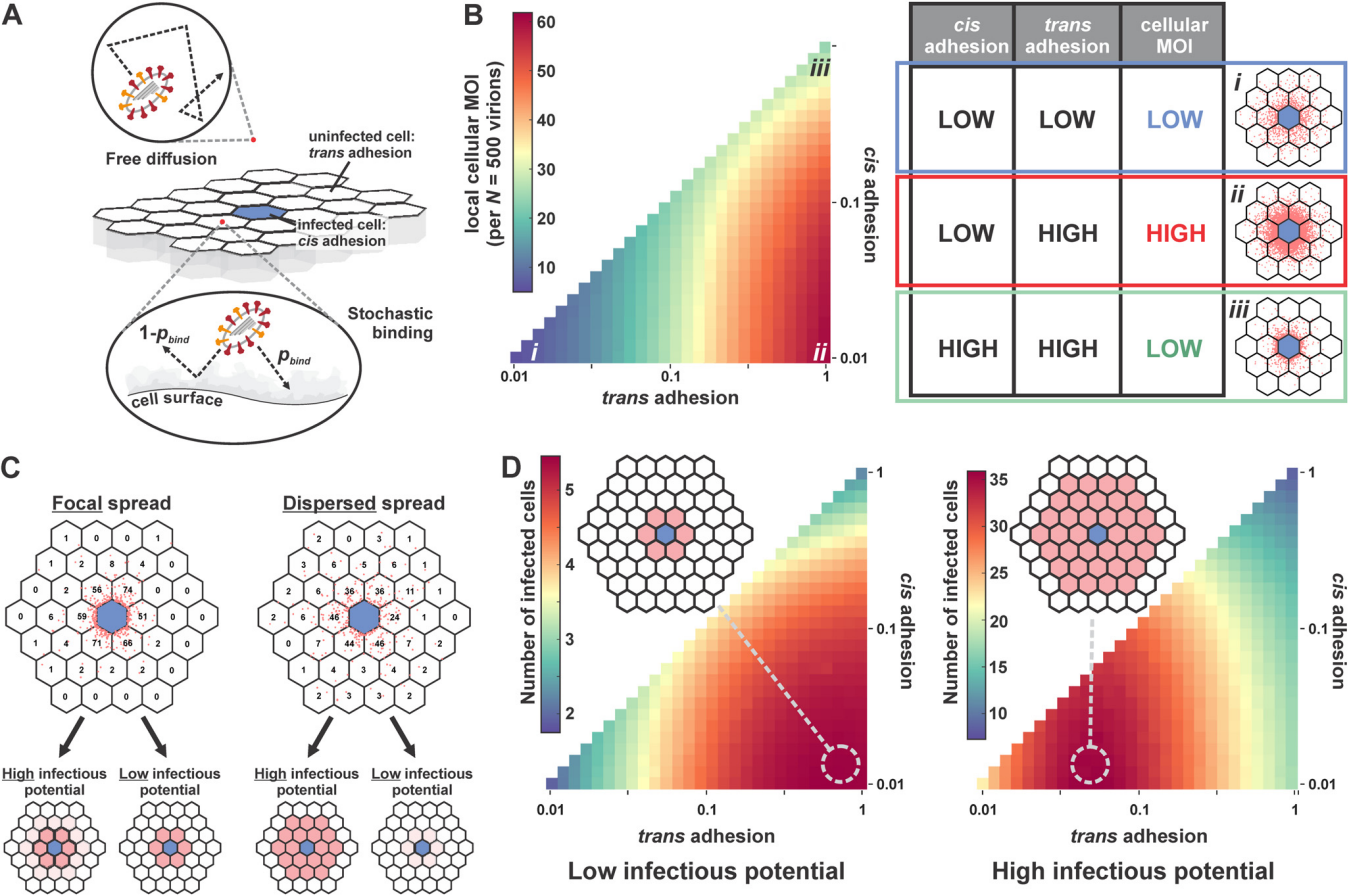
Optimal balance between IAV surface proteins depends on infectious potential. (A) Model of virus diffusion and binding to a cell monolayer. Virion attachment to the infected cell is referred to as “*cis* adhesion,” and attachment to uninfected cells is referred to as “*trans* adhesion.” During each encounter with the cell monolayer, the virions bind to the cell surface or get reflected with a probability (*p*_bind_), which differs for *cis* adhesion and *trans* adhesion. (B, left) Model predictions for the number of virions shed to neighboring cells (“local cellular MOI”) as a function of *cis* and *trans* binding probabilities. Numerical values correspond to a scenario in which the infected cell sheds a total of 500 virions. (Right) Summary of cellular MOI as a function of *cis* and *trans* adhesion probabilities. The images correspond to conditions labeled *i* to *iii* in the plot to the left. Blue hexagons (lattice center) correspond to the infected cell; red points denote the positions of bound virions. (C) Distribution of secondary infection as a function of viral adhesion (dispersed versus focal) and infectious potential (low versus high). Dispersed spread corresponds to a *trans* adhesion probability of 0.1, while focal spread corresponds to a *trans* adhesion probability of 1 (*cis* adhesion probability is 0.01 in both cases). For viruses with high infectious potential, dispersed spread leads to greater secondary infection, whereas focal spread produces more secondary infection when infectious potential is low. (D) Model predictions for secondary infection efficiency as a function of *cis* and *trans* adhesion. (Left) Viruses with low infectious potential (individual segment packaging probability of 80%, 5% infection-competent, burst size of 500) produce the greatest amount of secondary infection at high *trans* adhesion (~1), where virion spread is focal. (Right) Viruses with high infectious potential (individual segment packaging probability of 80%, 50% infection-competent, burst size of 500) produce the greatest amount of secondary infection at intermediate *trans* adhesion (~0.05), where virion spread is more dispersed.

Collectively, our results suggest a relationship between sialic acid availability, cellular MOI, and infectious potential, characteristics that will vary widely across IAV strains and their possible hosts. To broaden our investigation beyond the strains used in our experiments, we developed a simplified model that uses probabilistic virus attachment to simulate the spread of infection (Fig. 7A; Materials and Methods). We model viral attachment using two probabilities: a *cis* binding probability describes the likelihood that the virus will attach to the initial infected cell during a single random encounter, while a *trans* binding probability describes the likelihood of virus attachment to naive (i.e., nonparent) cells. These probabilities provide a framework for modeling the HA-NA functional balance. NA activity tends to drive the *cis* binding probability toward 0 and may also affect the *trans* binding probability, while HA binding avidity will affect both. This model provides estimates of cellular MOI and infection across a monolayer of cells, allowing us to determine how the surface features of a viral strain (i.e., its *cis* and *trans* binding probabilities) are functionally related to its dependence on coinfection (the number of virions necessary for infection).

Fig. 7B shows the predicted number of virions/cell adjacent to the initial site of infection across a 100-fold range in *cis* and *trans* binding probabilities (0.01 to 1). Not surprisingly, viral load per cell is highest when viruses bind strongly to naive cells with negligible binding to the infected cell but can be reduced when overall adhesion is either too weak or too strong (Fig. 7B, right). Predicted viral loads per cell can be used to estimate the probability of infection using our measured data for CA09 (Fig. 6C) or hypothetical parameters for viruses with a different dependence on coinfection. While strains requiring high viral loads for efficient infection favor focal spread in which *trans* binding probabilities are high (Fig. 7C and D, left), reduced *trans* binding is more efficient when fewer virions are necessary for infection (Fig. 7C and D, right). These predictions also generalize to viral burst size, with increased burst size permitting more dispersed spread for a given infectious potential and smaller burst sizes requiring more localized spread. Overall, these results predict that optimal HA-NA balance is functionally linked to intracellular aspects of viral replication, with potential implications for viral evolution and adaptation to new hosts.

## DISCUSSION

While the concept of HA-NA functional balance is well established, our work provides additional insights into how the competing activities of the IAV surface proteins shape the spatial structure of viral spread and the specific role of NA in this process. Depletion of sialic acid in both *cis* and *trans* (Fig. 3) combined with genetic differences in HA binding avidity (Fig. 4) collectively determine how virions spread from initial sites of infection (Fig. 5 to 7). HA and NA activities therefore constitute a genetic mechanism through which IAV may tune the degree of cellular coinfection that occurs during multicycle growth.

Our work reinforces previous observations that NA cleaves Sia both in *cis* and in *trans* and extends these observations to cell-surface NA with access to Sia on neighboring cells. The extent of cell-surface Sia depletion is similar when NA is expressed through infection or through transient transfection (Fig. 3D and G, bottom). This is perhaps surprising, given the potential differences in the level and duration of NA expression in these experiments. One possible explanation is that NA expressed at even modest levels is able to remove all Sia to which it has access. This may also be true for virion-associated NA, although we do not observe strong Sia depletion over the course of cellular entry (Fig. 3G, top). In this case, cell-surface Sia depletion may be restricted by the high rate at which virions are endocytosed following attachment (57). Further work is needed to determine how NA on the cell and viral surface separately contribute to virion release and dissemination and how the kinetics of Sia depletion influences the spread of virions produced earlier in the replication cycle (e.g., at ~6 to 8 h postinfection) versus those that are produced later.

The mechanistic link established here between the activities of viral surface proteins and the degree of cellular coinfection holds potential implications for virus evolution or adaptation to new hosts, in which a dependence on complementation is critical for virus replication (14). Specifically, our results suggest that strains that require higher degrees of coinfection or that produce smaller burst sizes within a particular host will spread most efficiently when adhesion to neighboring cells is strong, maximizing viral load per cell. While this comes at the cost of more dispersed spread, this may be beneficial to virus replication if dispersed virions are unlikely to result in productive infection. Previous work has demonstrated that the emergence of influenza viruses in new hosts is frequently accompanied by truncations in the NA stalk (58, 59). Although speculative, the link between reduced NA activity and increased cellular coinfection that we demonstrate here may provide insights into early events such as these during the adaptation of a virus to new hosts.

Additional mechanisms could further contribute to the frequency of coinfection, including transmission through tunneling nanotubes (60–62), as well as viral aggregation (63). Virion aggregation has been shown to promote coinfection by VSV and poliovirus (17, 64). In light of long-standing observations that IAV particles can form aggregates (65), it is plausible that aggregation could contribute to the spread of IAV. Importantly, aggregation-dependent coinfection could operate over long distances—potentially between hosts—and could involve multiple distinct IAV genotypes, produced by different infected cells. Understanding IAV phenotypes that contribute to aggregation-dependent coinfection could improve the current understanding of the constellation of viral factors that contribute to airborne transmission (66, 67).

Important aspects of IAV cellular spread remain, which the design of our study does not allow us to address. *In vivo* studies in animal models demonstrate that long-range transmission of viruses within the airways are rare but potentially very important events (9, 10). Given the limited number of virions released by a single infected cell and the high proportion of these that may attach to adjacent cells, be swept away by mucociliary clearance, or neutralized by antibodies, we speculate that pioneering rounds of focal infection (such as those studied here) may be necessary before long-range dispersal becomes favorable. Understanding how IAV strains differ in their dependence on focal versus dispersed spread in the human airways—and how host factors contribute to viral spread—will require additional work. The results presented here establish a framework for understanding the contributions of HA and NA to viral spread and cellular MOI in more complex environments.

## MATERIALS AND METHODS

### Cell lines and viruses.

Recombinant viruses were rescued using reverse genetics (68). Briefly, HEK-293T and MDCK-II cocultures were transfected with eight plasmids containing bidirectional promoters and encoding each viral genomic segment. Viral stocks were plaque purified and passaged at an MOI of ~0.001 in MDCK-II cells in virus growth medium comprised of Opti-MEM (Gibco), 2.5 mg/mL bovine serum albumin (Sigma-Aldrich), 1 mg/mL L-(tosylamido-2-phenyl ethyl) chloromethyl ketone (TPCK)-treated trypsin (Thermo Scientific Pierce), and 1× antibiotic-antimycotic (Corning). Infected cells were monitored for cytopathic effect and collected at early time points (~24 h). The RNA of rescued virus strains was extracted with QIAmp DSP viral RNA minikit (Qiagen), reverse transcribed, and amplified with OneTaq one-step reverse-transcription (RT)-PCR kit (NEB) for verification by Sanger sequencing.

The cell lines used in this study were purchased as authenticated cell lines (STR profiling) from ATCC and cultured under standard conditions (37°C, 5% CO_2_) using Dulbecco’s modified Eagle’s medium (DMEM) (Gibco) supplemented with 10% fetal bovine serum (FBS) (Gibco) and 1× antibiotic-antimycotic. A549 cells for measuring virion spread and for quantifying sialic acid were maintained in cell-growth medium. The cells were plated in an eight-chamber coverglass (Cellvis) coated with fibronectin (Sigma-Aldrich) 36 h prior to infection. After cells reached confluence, serum-containing medium was removed, and the cells were washed twice with phosphate-buffered saline (PBS) (pH 7.4) (Gibco) before adding viral stocks diluted to MOI 0.003 in virus growth media. After 1 h of incubation at 37°C, the cells were washed with PBS and the virus-containing medium was replaced with fresh virus growth media containing fluorescent Fab fragments to monitor single or multiple round infection. For the experiments shown in Fig. 6, an additional 0.2 μg/mL TPCK-treated trypsin was included in the medium to permit multiround replication.

### Isolation and culture of primary human tracheal epithelial cells.

The use of human tracheobronchial epithelial cells (HTECs) isolated from surgical tissue was exempted from regulation as human subject research by the Institutional Review Board of Washington University in St. Louis. Epithelial cells were obtained from excess tracheobronchial airways of lungs donated for transplant. Airway progenitor cells were isolated and cultured as previously described (69). In brief, airway epithelial cells were released from airway tissues following incubation in pronase and then expanded on rat tail collagen-coated plastic culture dishes in custom medium. The cells were subcultured on collagen-coated Transwell-supported membranes (Corning) using medium containing 2% Nuserum (Corning). Once cells were confluent, the apical medium was removed to create an air-liquid interface that was used to induce differentiation to secretory and multiciliated cells. The cells were used for influenza virus infection after at least 4 weeks of air-liquid interface culture.

### Quantification of cell-surface sialic acids.

Cell-surface sialic acids were labeled using aniline-catalyzed oxime ligation (41). In comparison to labeling with Sambucus nigra lectin (SNA) (Vector Laboratories), which are large and have defined preferences for specific Sia linkages, chemical labeling provides a more reproducible and quantitative measurement of cell-surface sialic acids (Fig. S5). To perform this reaction, we first prepared solution A (1 mM NaIO_4_ [Sigma-Aldrich] dissolved in PBS supplemented with 1 mM CaCl_2_) and solution B (1 mM CaCl_2_, 10 mM aniline [Sigma-Aldrich], 5% FBS and 100 μM CF633 hydrazide [Sigma-Aldrich], or CF488A hydrazide [Sigma-Aldrich] in cold PBS, pH 6.5 [Teknova]). The cells were cooled on ice and washed with cold PBS once before incubating with solution A on ice for 15 min, followed by a wash with cold PBS (pH 6.5) and incubation with solution B on ice for 40 min. The cells with labeled sialic acids were washed once with cold Opti-MEM, and their plasma membranes were labeled with CellMask Orange (Invitrogen) at a concentration of 2.5 μg/mL in Opti-MEM for 5 min at room temperature. The cells were washed again with Opti-MEM before imaging.

### Quantification of NA activity with MUNANA.

HEK-293T cells were transfected with pCAGGS plasmids containing NA sequences from WSN33, CA09, HK68, and BR07 with C-terminal Myc tags attached via a short flexible linker (GGSEQKLISEEDL). Cells transfected with Poly(ethylenimine) (PEI) (Polysciences) were incubated for 48 h at 33°C. The medium was then removed, and the cells were washed once with PBS (pH 7.4), suspended by pipetting, and serially diluted in PBS into a 96-well glass-bottomed plate (Cellvis) as a 50-μL suspension. A total of 50 μL of NA buffer (100 mM NaCl, 50 mM morpholineethanesulfonic acid [MES], pH 6.5, 5 mM CaCl_2_, and 5% bovine serum albumin) supplemented with 0.25 mM 2′-(4-methylumbelliferyl)-α-d-*N*-acetylneuraminic acid (MUNANA) sodium salt (Toronto Research Chemicals) was then added to each well. The plate was incubated at 37°C for 1 h before adding 100 μL stop solution (150 mM NaOH in 83% ethanol). MUNANA signal was measured with a Nikon Ti2 confocal microscope using excitation at 405 nm. In parallel with MUNANA tests, 10 μL of transfected cells from each sample were plated for imaging with the anti-myc antibody 9E10 (70) labeled with Sulfo-Cy5. Cell-surface NA expression was quantified from confocal stacks (1-μm step, collected with a 40×, 1.30-NA objective) and used to normalize activities determined from MUNANA measurements.

### Quantifying local virus shedding in A549 monolayers.

Infected cells (MOI ≈ 0.003) were imaged with a Nikon Ti2 confocal microscopy system using a 40×, 1.30-NA objective at 16 h.p.i. Infected cells were selected based on surface expression of HA (using 9 nM CR9114 Fab for H1N1 strains and 19 nM FI6v3 plus 19 nM H3v-47 [71] for H3N2 strains) and M2 (using a Fab fragment derived from mAb148 [72]). Confocal z-stacks spanning a range of 15 μm were collected for the channel corresponding to labeled HA. Image analysis was performed on Nikon NIS Element software 5.21. Briefly, a maximum intensity projection was generated from confocal *z*-stacks, from which the body of the infected cell was identified by size and intensity and stored (Mask A). This mask was dilated by ~1 cell diameter to capture a concentric region surrounding the infected cell (Mask B). Peak detection was performed within the region between Masks A and B, where bright spots corresponding to shed virions were identified and counted.

### Visualizing local virus shedding in differentiated HTECs.

The apical surfaces of HTECs grown in ALI culture for >28 days were washed twice with PBS prior to infection with A/Brisbane/10/2007 (H3N2) or A/California/04/2009 (H1N1) virus at an MOI of ~0.01 to 0.1. Virus for infections was diluted into 20 μL of PBS added to the apical compartment for 1 h at 37°C. Following infection, excess virus was removed, and the apical compartment was washed with PBS and left dry. At 8 h.p.i., 100 μL PBS containing fluorescent CR9114 Fab (10 nM) was added to the apical compartment and removed without further washing. The cells were then returned to the incubator until 16 h.p.i., when they were cooled on ice, washed with ice-cold PBS, and fixed using 4% paraformaldehyde. Fixed cells were permeabilized and labeled overnight at 4°C using the antiacetylated α-tubulin antibody 6-11B-1 (Santa Cruz Biotech) at 0.4 μg/mL in fetal bovine serum. Immediately prior to imaging, the cells were washed with PBS, and the Transwell inserts were excised with a razorblade and mounted face down on a coverslip for imaging with a Ti2 inverted confocal microscope with a 60×, 1.40-NA objective.

### Quantifying virion shedding to the supernatant.

The cells were infected at an MOI of ~0.2 and 9 nM fluorescently labeled CR9114 Fab was added at 1 h.p.i. to restrict the infection to a single round. At 16 h.p.i., the supernatant was collected and spun onto A549 cell monolayers grown in eight-well chambered coverglass at 2,500 × *g* for 20 min. The z-stacks of the A549 monolayer (16-μm total range, 1-μm step size) were imaged with a Nikon Ti2 confocal microscopy system using a 40×, 1.30-NA objective. The projection images containing virus particles from the z-stacks were then analyzed using spot detection to determine the total number of virions within the well. To normalize the virus particle counts, the number of infected cells from which this virus was produced was determined at the time of virus collection using a 10×, 0.45-NA objective. The total number of infected cells/well was calculated by adding up the products of the ratio of infected cells and the total number of cells/well, determined by labeling cell nuclei with Hoechst 33342 (Thermo Scientific Pierce), among 56 fields of views that cover the entire well. To evaluate the proportion of virus that was captured by centrifugation onto A549 monolayers, concentrated viral stocks were subjected to centrifugation as above, and viral content in the supernatant before and after centrifugation was evaluated via quantitative Western blot with an anti-HA antibody PA5-34929 (Invitrogen) (Fig. S3B).

### Antibody purification and labeling.

VH and VL sequences of Fabs were obtained from deposited antibody structures and cloned into backbones containing the CH1 and CL domains, respectively. Heavy chain Fab sequences were modified with a C-terminal ybbR tag for enzymatic labeling (73) and a His_6_ tag for affinity purification. HEK-293T cells at ~85% confluency were washed with PBS, transfected with verified clones, and grown in Opti-MEM with 2% FBS for 7 days. Supernatants were collected and purified using Ni-nitrilotriacetic acid (NTA)-agarose (Thermo Scientific HisPur). Eluted antibodies were quantified by UV-visible spectroscopy, diluted into a new buffer for enzymatic labeling (150 mM NaCl, 25 mM HEPES, 5 mM MgCl_2_), and concentrated to ~1 mg/mL by Vivaspin 20 centrifugal filter unit (molecular weight cutoff [MWCO], 10 kDa) (Sartorius). Sfp synthase and CoA-conjugated dyes were prepared as previously described (56) and used to perform the overnight Fab labeling reaction on ice. Excess dye was removed by PD-10 desalting columns (Cytiva). Expression and purification of full-length IgG1 antibodies (9E10 and 1G01 [74]) followed a similar procedure, except using serum-free medium for expression and protein A/G-agarose (Thermo Scientific Pierce) for affinity purification.

### Creating polyclonal A549 SLC35A1 knockout cells.

To evaluate the degree of nonspecific Sia labeling, we generated A549 knockout cells lacking the CMP sialic acid transporter SLC35A1, essential for the surface expression of IAV receptors (75). A549 knockout cells were generated through transduction with lentivirus generated from the lentiCRISPR v2 packaging plasmid. Three single guide RNA (sgRNA) sequences were selected using CRISPR KO and the design rules described by Doench et al. (76). These were tested in small scale via transient transfection in HEK-293Ts, and the sgRNAs that yielded the highest efficiency (determined by measuring the loss of cell surface Sia) were selected for lentivirus preparation and infection into A549s. The optimal spacer sequence was 5′-GACAGTGCATAAAGCAGTACA-3′ (underlined nucleotide added for efficient transcription initiation).

### Measuring virus binding avidity.

To measure virion binding avidity, A549 cells with different Sia abundance were prepared by treatment with different concentrations of *Cp*NA (Roche) for 30 min at 37°C. Simultaneously, viruses were labeled with fluorescent Fab fragments (18 nM CR9114 for H1N1, 19 nM FI6v3 plus 19 nM H3v-47 for H3N2) for 20 min at room temperature. Following *Cp*NA treatment, the cells were washed with PBS twice and incubated with 100 μL virus-containing Opti-MEM at 4°C. After incubating for 30 min, virus-containing medium was removed, and the cells were washed and supplemented with cold Opti-MEM. Viruses attached to the cell surface or endocytosed were imaged by the Nikon Ti2 confocal microscopy system using a 40×, 1.30-NA objective.

### Measuring infectious potential.

To measure the relationship between viral particles and the probability of infection, we prepared stocks of CA09 virus in A549 cells. Viruses were concentrated approximately 5-fold by centrifugation at 21,100 × *g* for 30 min at 4°C. Concentrated virus was then serially diluted and added to A549 cell monolayers. After 30 min of incubation at 37°C, the cells were washed with PBS twice and supplemented with virus growth medium containing 9 nM labeled CR9114 Fab. To quantify the number of bound virions for each group, the same concentrations of viruses preincubated with 9 nM CR9114 Fab for 20 min were added to another group of cells and incubated for 30 min before washing off. Particle numbers were obtained by collecting confocal stacks and performing particle detection based on the maximum intensity projection. For the group incubated with untreated virus, the percentage of infected cells was determined by measuring the ratio of HA-positive area to the total field of view (containing an intact cell monolayer) at ~12 h.p.i.

### Modeling infectious potential.

To interpret our measurements of infection probability versus the average number of particles/cell, we developed a model that accounts for (i) virion delivery of incomplete genomes (i.e., semi- or fully infectious particles); (ii) virions that fail to deliver any genome segments (i.e., noninfectious particles); and (iii) the Poissonian nature of virion attachment to cells. We also outline how this could be extended to subpopulations of cells that differ in their susceptibility to infection. Importantly, particles considered noninfectious are not necessarily inherently defective but may have failed to deliver genomic segments due to random chance. Similarly, some particles considered semi-infectious within this model may be inherently defective/defective interfering. Because our experimental readout does not allow us to differentiate between these possibilities, we restrict our model to fully infectious, semi-infectious, and noninfectious virions.

### (i) Incomplete genomes.

We define an eight-element vector, *p*, whose elements (*p_i_*) correspond to the probabilities that a competent virion delivers segment *i* to the target cell (Fig. S8A). While each probability *p_i_* could be distinct, for simplicity, we model the probabilities as being equal across all segments. Since our data capture only cells that express HA, we assume that these cells express a minimum of five genomic segments: the vRNP segments (NP, PA, PB1, and PB2), along with the HA segment. This assumption is motivated by previous reports showing that secondary transcription (i.e., transcription from nascent vRNPs) is necessary for robust expression of other segments (12).

### (ii) Noninfectious virions.

Some fraction of virions within a population will not be capable of contributing to infection. These particles are distinct from those that enter the cells but package or deliver an incomplete genome. These particles could arise from failure to package any genomic segments (empty particles) or from failure to escape from the endosome (e.g., due to incomplete proteolytic activation of HA or for other reasons). To account for noninfectious virions, we define a parameter, ϕ, that describes the fraction of the viral population that contributes to infection.

### (iii) Virion attachment to cells.

Our measurements of particles/cell represent a population average that does not apply to any one particular cell within the population. To account for this, we model the distribution of particles/cell as following a Poisson distribution. If the average number of particles/cell is *N*, and the fraction of these that are competent for infection is ϕ, then the distribution of infection-competent virions/cell (*n*) will follow:

$$f_{1}\left(n\right)=\frac{{\left(\phi N\right)}^{n}e^{-\phi N}}{n!}$$

From this relationship and the segment delivery probabilities for competent virions, we can calculate infection probabilities. First, we note that the probability of failing to deliver segment *i* in *n* tries is equal to ${\left(1-p_{i}\right)}^{n}$. Therefore, the probability of successfully delivering at least one copy of segment *i* in *n* tries is equal to $1-{\left(1-p_{i}\right)}^{n}$. Cell-surface expression of HA indicates delivery of at least one copy of at least five segments (NP, PA, PB1, PB2, and HA), the probability of which is equal to the product of the individual segment delivery probabilities:

$$f_{2}(n)=\overset{5}{\underset{i=1}{\prod }}1-{\left(1-p_{i}\right)}^{n}$$

Combining the two probabilities from above, we can determine the probability of infection for a sample in which the average number of particles/cell is *N*:

$$f\left(N\right)=\overset{\infty }{\underset{n=0}{\sum }}f_{1}(n)f_{2}(n)=\overset{\infty }{\underset{n=0}{\sum }}\frac{{\left(\phi N\right)}^{n}e^{-\phi N}}{n!}\left[\overset{5}{\underset{i=1}{\prod }}1-{\left(1-p_{i}\right)}^{n}\right]$$

This equation can then be used to interpret our experimental measurements. In particular, this provides a means of estimating the fraction of competent virions within our sample, as well as the segment delivery probabilities (Fig. 6C; Fig. S8).

### (iv) Differences in cellular susceptibility.

Cells within a population may differ in their susceptibility to infection. This could manifest as differences in segment delivery probabilities (*p_i_*) or differences in infection competence (ϕ) for particular subpopulations of cells. This model can be extended to account for these differences by calculating different infection probabilities for each subpopulation of cells and weighting these probabilities by the relative size of each subpopulation to determine the net probability of infection. For simplicity, our analysis focuses on the case in which each cell has equal susceptibility to infection. Including multiple subpopulations of cells does not appreciably improve the fit to CA09 data in Fig. 6C and Fig. S8.

### Modeling virion spread.

We modeled the spread of virions from infected cells as a three-dimensional random walk above a cellular monolayer, represented by a partially absorbing boundary. Cells within the monolayer are represented by hexagons in a lattice, whose centers are separated by 30 μm. Virions are released synchronously from a central hexagon (the infected cell) and sample a random displacement with a mean of 0 and a standard deviation of 3 μm in each direction for every time step (=1s) of the simulation. These values correspond to a diffusion coefficient of ~5 μm^2^/s, consistent with predictions from the Stokes-Einstein equation for a virion with a diameter of 100 nm in water. When a virion encounters the cell monolayer, it either binds to the surface irreversibly (with the probability *p*_bind_) or reflects from the surface to continue its random walk (with the probability $1-p_{\text{bind}}$), potentially encountering the surface repeatedly over time. We assign distinct binding probabilities to the surface of the infected cell (*cis* adhesion) and to uninfected cells (*trans* adhesion). We simulate only conditions under which the *cis* adhesion probability is less than or equal to the *trans* adhesion probability, reflecting the efficient removal of Sia in *cis* during infection (Fig. 3). The output of the model is a spatial distribution of bound and unbound virions, which we truncate after 8 h of simulated time (28,800 iterations; additional simulations extended to 16 h yield quantitatively similar results, with an ~5% increase in the number of infected cells when adhesion is low and infectious potential is high). Using this spatial distribution, we determine the cellular MOI and the probability of infection for each cell that falls within a 25 × 25 hexagonal lattice (~650 μm × 800 μm in size). For viruses that attach outside the defined lattice, we calculate the probability of infection using a single-shot model (i.e., the product of the segment packaging probabilities *p_i_* multiplied by the fraction ϕ that is infection-competent); this is motivated by the extremely low probability of coinfection at these distances when the burst size ≤1,000. The expected number of infected cells is then determined by summing the probabilities for individual cells. The results of this analysis are shown in Fig. 7 using estimates of infectious potential measured for CA09 (Fig. 7D, left) or for virions with hypothetical characteristics (Fig. 7D, right).

### Statistics and replicates.

Replicates referenced throughout the paper refer to biological replicates, defined as separate cultures of cells infected/transfected/treated individually and assayed as indicated. All statistical tests were performed in Python Scipy 1.7.3. No statistical methods were used to predetermine sample size. The statistical tests and the number of replicates used in specific cases are described in the figure legends. Box plots may sometimes not show the outliers due to the limitation of the *y* axis.

### Data availability.

The codes for modeling infectious potential and virion spread are available at https://github.com/mvahey/2023NA.
