# Supplementary material for: Neuraminidase Activity Modulates Cellular Coinfection during Influenza A Virus Multicycle Growth
Source: mBio. 2023 Apr 20;14(3):e03591-22. doi: 10.1128/mbio.03591-22 (PMC10294670; doi:10.1128/mbio.03591-22)
Supplement: FIG S1 [file mbio.03591-22-s0001.pdf]

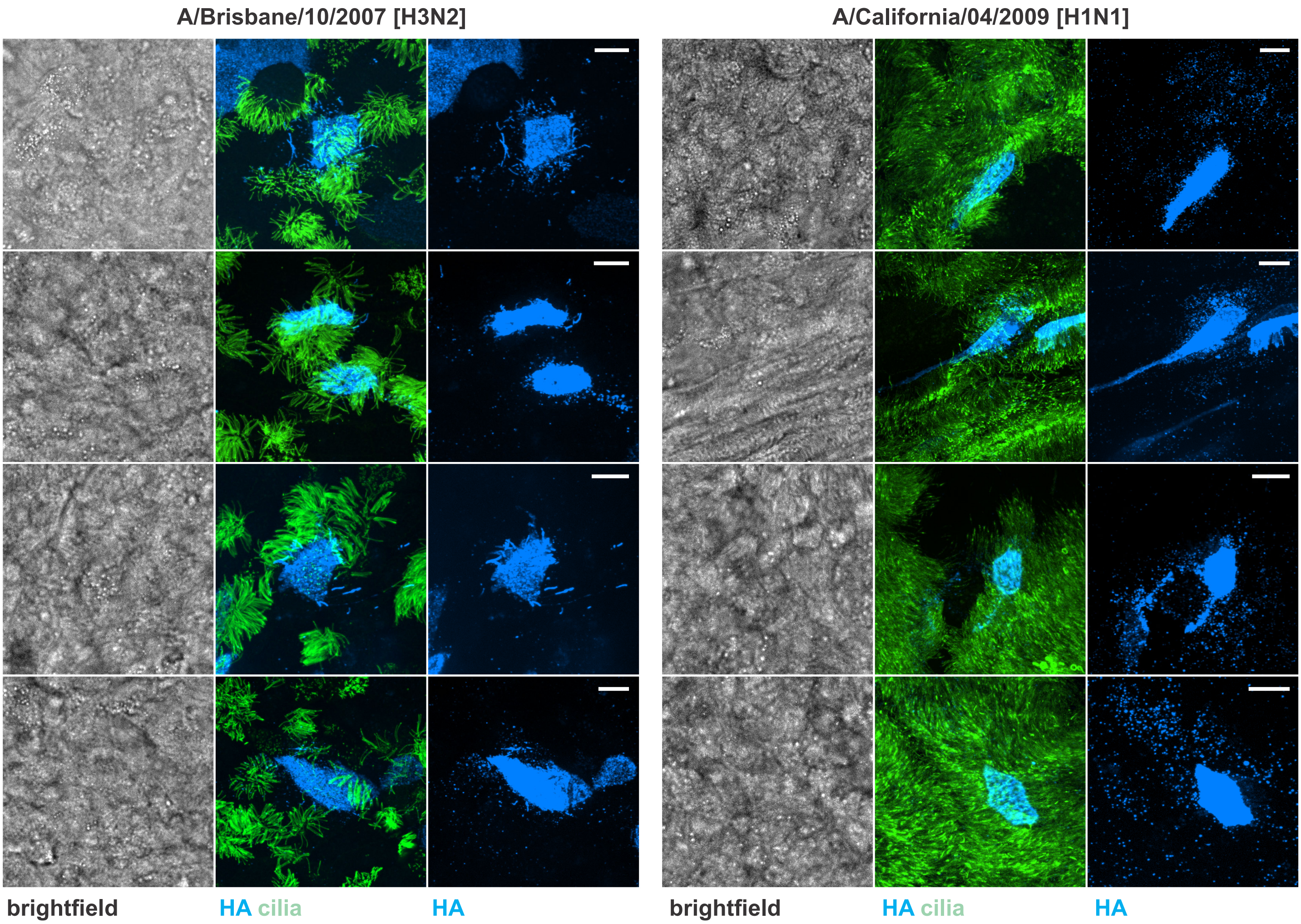

Figure S1: Local virion spread in differentiated human tracheal cells.

Images of differentiated HTECs infected with different strains of IAV and imaged following fixation at 16 h.p.i. HA is labeled with CR9114 Fab; cilia are visualized with an anti-acetyl tubulin antibody conjugated with Alexa Fluor 488. Contrast in the HA channel is exaggerated in the rightmost panels to make shed virions visible. Scale bars = 10 µm.
