# Supplementary material for: Neuraminidase Activity Modulates Cellular Coinfection during Influenza A Virus Multicycle Growth
Source: mBio. 2023 Apr 20;14(3):e03591-22. doi: 10.1128/mbio.03591-22 (PMC10294670; doi:10.1128/mbio.03591-22)
Supplement: FIG S2 [file mbio.03591-22-s0002.pdf]

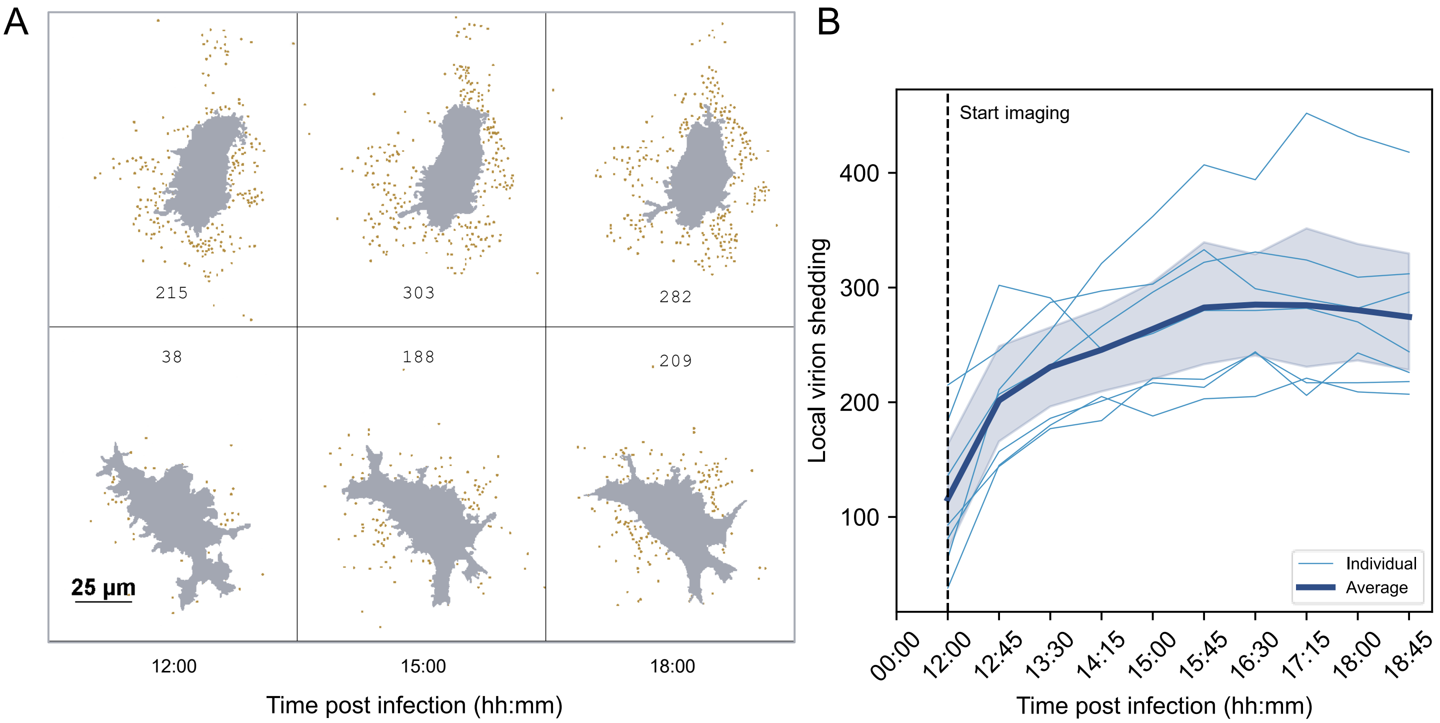

Figure S2: Progression of virion shedding to neighboring cells over time.

(A) Monitoring local virion shedding in A549 cells infected by WSN33. Confocal stacks of infected cells are taken starting from 12 h.p.i. Gray region marks the cell body, with detected virions highlighted in gold. Cells selected for analysis show expression of both HA and M2 on the cell surface.

(B) Quantification of local virion shedding compiled from time series of seven cells infected by WSN33. Shaded region represents 95% confidence interval.
