# Supplementary material for: Neuraminidase Activity Modulates Cellular Coinfection during Influenza A Virus Multicycle Growth
Source: mBio. 2023 Apr 20;14(3):e03591-22. doi: 10.1128/mbio.03591-22 (PMC10294670; doi:10.1128/mbio.03591-22)
Supplement: FIG S3 [file mbio.03591-22-s0003.pdf]

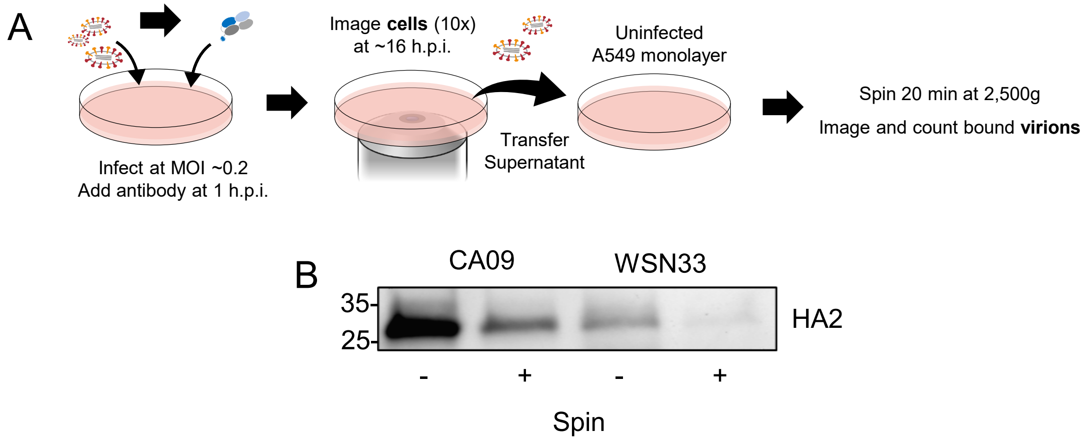

Figure S3. Quantification of viral release into the culture supernatant.

(A) Schematic showing the experimental procedure. Cells are infected (MOI ~0.2) and labeled with fluorescent Fab starting at 1 h.p.i. Virus is collected at 16 h.p.i. and bound under centrifugation to the surface of a fresh A549 monolayer for quantification by confocal microscopy.

(B) Quantification via Western blot of virus remaining in the supernatant following centrifugation onto A549 monolayers as in (A). 23% and 21% virions remain after spinning for WSN33 and CA09 respectively, suggesting that we are able to detect the majority of virions released into the supernatant.
