# Supplementary material for: Neuraminidase Activity Modulates Cellular Coinfection during Influenza A Virus Multicycle Growth
Source: mBio. 2023 Apr 20;14(3):e03591-22. doi: 10.1128/mbio.03591-22 (PMC10294670; doi:10.1128/mbio.03591-22)
Supplement: FIG S4 [file mbio.03591-22-s0004.pdf]

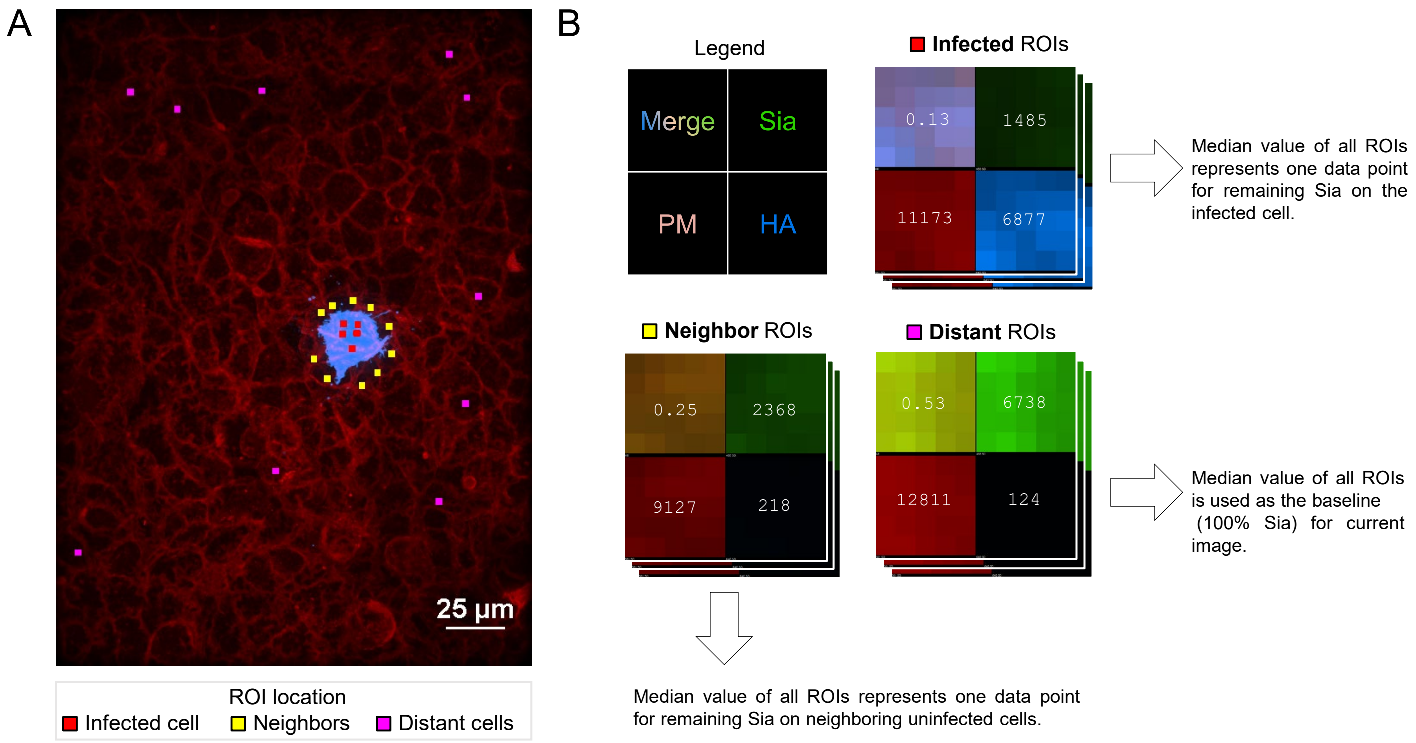

Figure S4: Quantifying depletion of cell-surface Sia using hydrazide coupling.

(A) Image showing regions of interest (ROIs) sampled from the surface of an infected cell (HA+), uninfected neighbors, and uninfected distant cells.

(B) Enlarged ROIs in split view. The number on each split channel represents the mean intensity. The number on "Merge" channel represents the Sia signal normalized by the plasma membrane ('PM') signal, proportional to Sia per unit membrane area. The size of each ROI is 0.55 µm by 0.55 µm.
