# Supplementary material for: Neuraminidase Activity Modulates Cellular Coinfection during Influenza A Virus Multicycle Growth
Source: mBio. 2023 Apr 20;14(3):e03591-22. doi: 10.1128/mbio.03591-22 (PMC10294670; doi:10.1128/mbio.03591-22)
Supplement: FIG S5 [file mbio.03591-22-s0005.pdf]

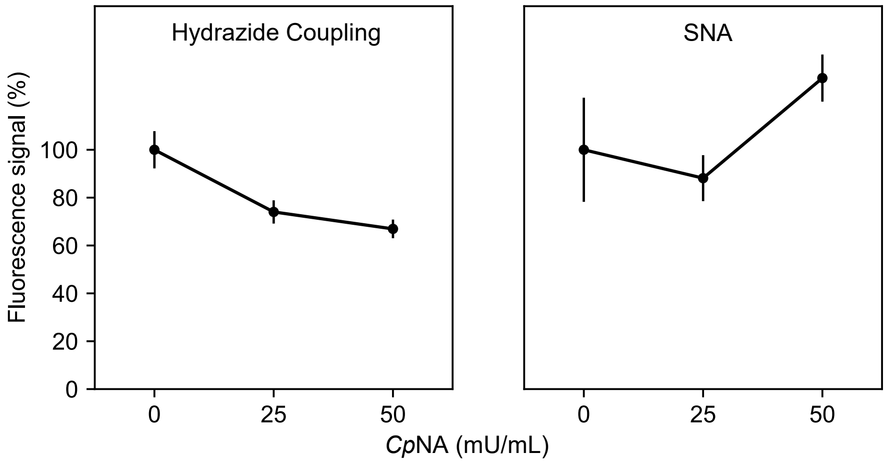

Figure S5: Hydrazide conjugation sensitively and reproducibly reports Sia depletion by exogenous sialidase.

Quantification of Sia levels on A549 cell monolayers treated with indicated concentrations of *Cp*NA for 30 min at 37 °C using hydrazide coupling (left) and lectin labeling (SNA; right). Points represent mean values of three individual experiments (two for untreated group). Error bars show standard deviation.
