# Supplementary material for: Neuraminidase Activity Modulates Cellular Coinfection during Influenza A Virus Multicycle Growth
Source: mBio. 2023 Apr 20;14(3):e03591-22. doi: 10.1128/mbio.03591-22 (PMC10294670; doi:10.1128/mbio.03591-22)
Supplement: FIG S6 [file mbio.03591-22-s0006.pdf]

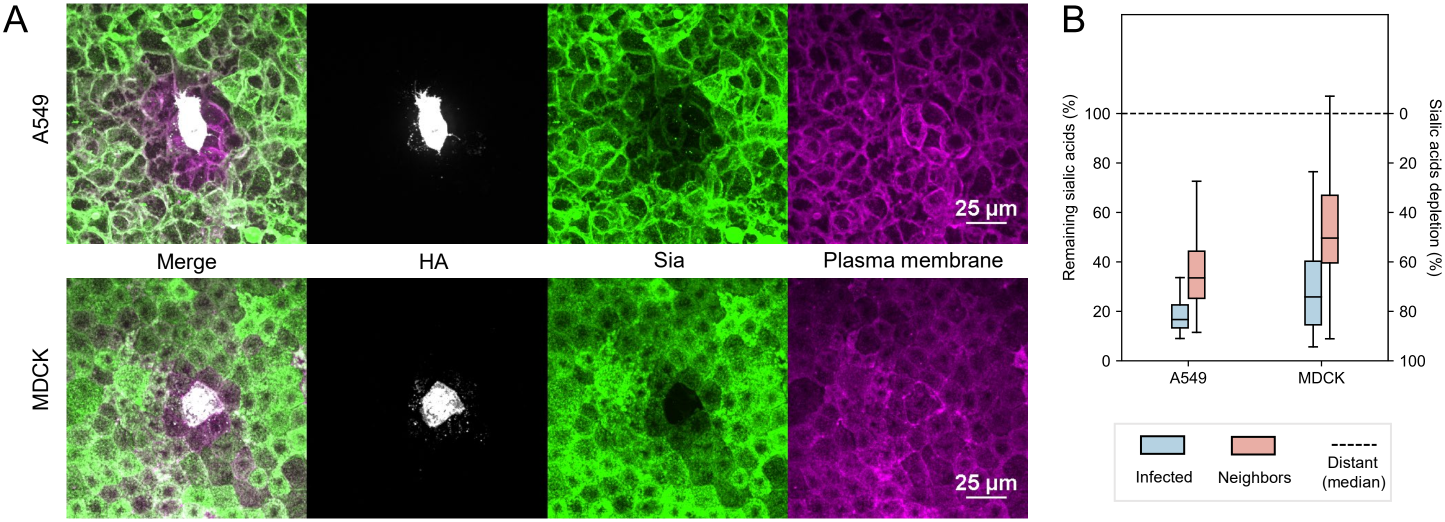

Figure S6: *Cis* and *trans* depletion of Sia is observed following infection of multiple cell types.

(A) Images showing HA, Sia and plasma membrane in the proximity of cells infected with CA09 at MOI of 0.003.

(B) Quantification of remaining Sia on infected and neighboring cells for A549 and MDCK cell lines. Data is from at least 17 sites of infection per cell line.
