# Supplementary material for: Neuraminidase Activity Modulates Cellular Coinfection during Influenza A Virus Multicycle Growth
Source: mBio. 2023 Apr 20;14(3):e03591-22. doi: 10.1128/mbio.03591-22 (PMC10294670; doi:10.1128/mbio.03591-22)
Supplement: FIG S7 [file mbio.03591-22-s0007.pdf]

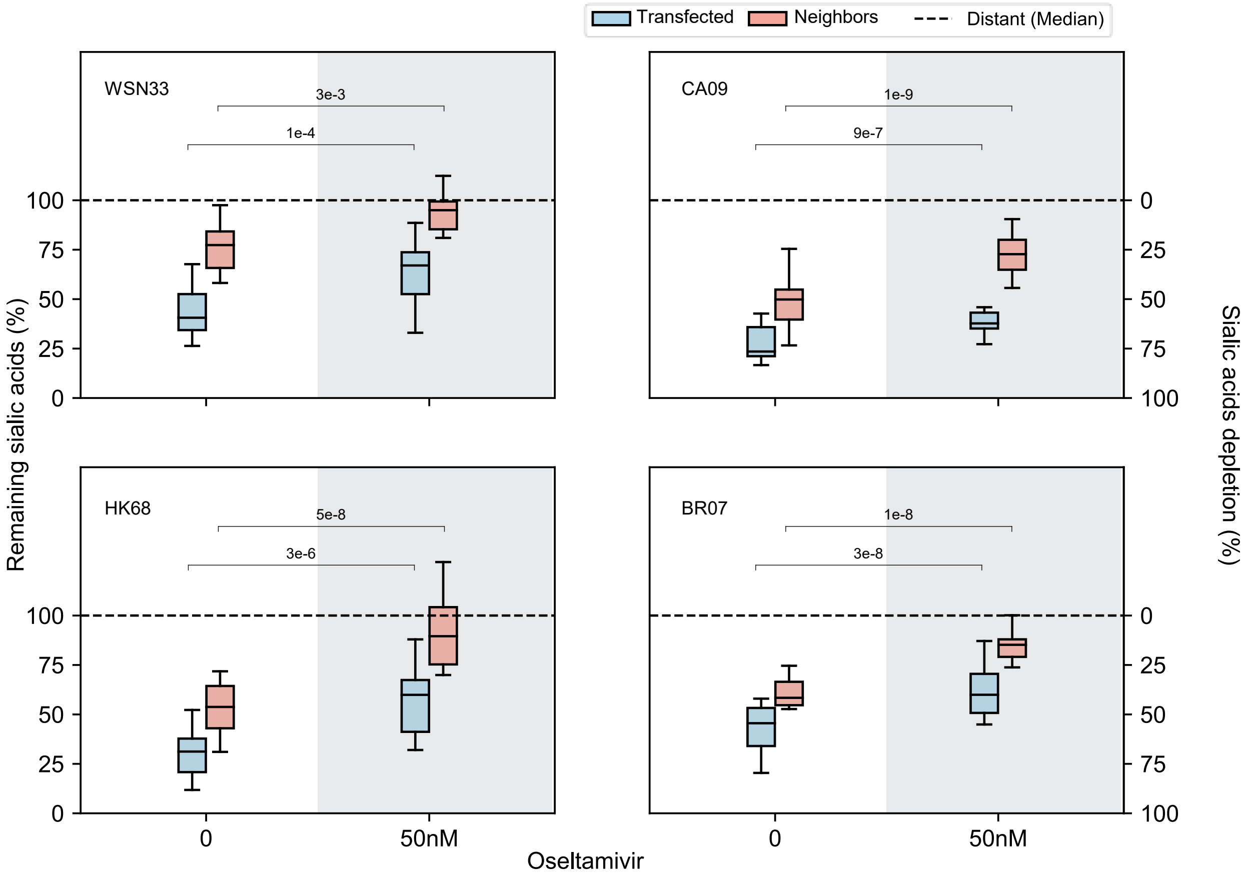

Figure S7. Quantification of Sia depletion under oseltamivir treatment.

Cells were incubated for 48 h at 33 °C after WSN33, CA09, HK68 and BR07 NA transfection. For oseltamivir groups, 50 nM oseltamivir was added at the time of transfection. All treatments were performed side-by-side for comparison and contain data from at least 10 cells. P-values are determined by independent t-tests.
