# Supplementary material for: Neuraminidase Activity Modulates Cellular Coinfection during Influenza A Virus Multicycle Growth
Source: mBio. 2023 Apr 20;14(3):e03591-22. doi: 10.1128/mbio.03591-22 (PMC10294670; doi:10.1128/mbio.03591-22)
Supplement: FIG S8 [file mbio.03591-22-s0008.pdf]

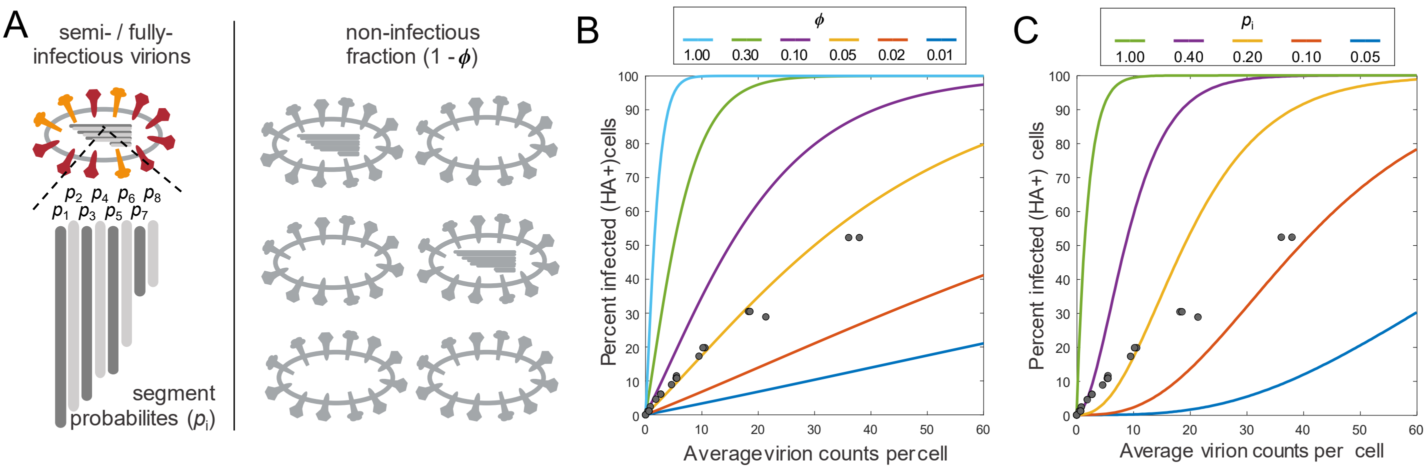

Figure S8: Modeling virus infection.

(A) Illustration of virus populations represented by the model. Semi- or fully- infectious particles can have different probabilities of delivering each segment. The proportion of the virus population that contributes to infection is given by ϕ.

(B) Predictions from the infection model for different proportions of non-infectious virions $(1-\phi)$ with $p_\mathrm{i} = 0.8$ (*i.e.*, an infectious virus delivers any particular genome segment with an 80% probability). Results are shown for the percentage of HA+ cells. Fitting CA09 infectivity data suggests values of $p_\mathrm{i} = 0.8$ and $\phi = 0.05$.

(C) Predictions from the infection model for different segment delivery probabilities p_i where $\phi = 0.5$. ‘s’ shape of curves with lower p_i values do not recapitulate data.
